# ﻿Preliminary checklist of the genus *Festuca* L. (Loliinae, Pooideae, Poaceae) in the Altai Mountains with outlines for further studies

**DOI:** 10.3897/phytokeys.234.105385

**Published:** 2023-10-26

**Authors:** Polina D. Gudkova, Elizaveta A. Kriuchkova, Alexander I. Shmakov, Marcin Nobis

**Affiliations:** 1 Research laboratory ‘Herbarium’, National Research Tomsk State University, Lenin 36 Ave., 634050 Tomsk, Russia Altai State University Barnaul Russia; 2 Department of Botany, Institute of Biology and Biotechnology, Altai State University, Lenin 61 Ave., 656049 Barnaul, Russia National Research Tomsk State University Tomsk Russia; 3 Institute of Botany, Faculty of Biology, Jagiellonian University, Gronostajowa 3, 30-387 Kraków, Poland Jagiellonian University Kraków Poland

**Keywords:** Distribution, *Festucaovina* group, fine-leaved fescue, identification key, taxonomic revision

## Abstract

Here we present an updated checklist of the genus *Festuca* in the Altai Mountains (AM). The study was carried out on the abundant herbarium material and considered the latest published phylogenetic analyses. *Festuca* was revised within the scope of the fine-leaved group (clade) with two sections, sect. Aulaxyper and sect. Festuca. Two species, namely *F.richardsonii* and *F.lenensis*, were previously misidentified and are not present in the AM. *Festucabrevissima* is a new record for the Russian part of the AM and for the flora of Mongolia. In total, our revision shows that 17 species of fine-leaved fescues are present in the area of AM. In this paper, we provide a key to species identification, as well as illustrations of plants, habits, leaves, spikelets, and glumes. Information on nomenclature types, synonymy, flowering period, chromosome numbers, habitats, and general distribution along with distribution maps of the particular species within the AM are included.

## ﻿Introduction

The fescue genus, *Festuca* L., is one of the largest genera of the Poaceae family and includes more than 600 species with the greatest diversity in the Holarctic zone of Eurasia and North America (Tzvelev 1976; Alexeev 1980; Clayton and Renvoize 1986; Aiken and Darbyshire 1990; Watson and Dallwitz 1992; Soreng et al. 2003, 2022; Сlayton et al. 2006; Lu et al. 2006; Stančik and Peterson 2007). The genus is easily recognisable by its perennial caespitose or rhizomatous plant form, paniculate inflorescences, 3–11-floret spikelets, dorsally rounded lemmas with 3–5 veins, a linear hilum, and lack of a hairy appendage on the ovary apex (Kellogg 2015; Ospina-González et al. 2015). A base chromosome number of the genus *Festuca* is *x*=7 (Ospina-González et al. 2015). However, fescues are often polyploids, and even representatives of one species may be characterised by a different number of chromosomes (Šmarda and Kočí 2003; Šmarda et al. 2005; Galli et al. 2006; Enushchenko and Probatova 2020; Kiedrzyński et al. 2021). In the Altai Mountains (AM), the fescues are diploids, tetraploids or hexaploids. Different ploidy level is typical for species such as *F.rubra* L., *F.kryloviana* Reverd., *F.pseudovina* Hach. ex Wiesb., *F.lenensis* Drobow and *F.valesiaca* Schleich. ex Gaudin (Mizianty and Pawlus 1984; Alexeev et al. 1988; Šmarda 2008; Dirihan et al. 2016; Tzvelev and Probatova 2019). It is considered that ploidy level influences the morphological characters including the diagnostic ones, such as the length of the lemma, the length of the spikelet, the width of the vegetative leaves in the way that higher ploidy levels results in generally larger sizes of the particular parts of the plants (Litardière 1923; Levitskii and Kuzmina 1927; Bidault 1968; Rewicz et al. 2018). Therefore, the species whose representatives are characterised by polyploidy are highly polymorphic, which significantly complicates species identification. In the Altai Mountains, the fescues are diploids, tetraploids or hexaploids. Although fescues are widely distributed in the steppe zone and have regional economic importance as forage grasses, due to taxonomic problems and challenging species identifications, the genus is understudied, and thus all the more important (Torrecilla and Catalán 2002; Torrecilla et al. 2003, 2013; Ospina-González et al. 2015; Bednarska and Brazauskas 2017; Ospina and Picca 2019; Enushchenko and Probatova 2020; Boeuf et al. 2022; Vasile et al. 2022).

The Altai Mountains (AM) are located in Russia, Kazakhstan, Mongolia and China. The AM host 2700 species of vascular plants, with Poaceae being one of the most widely distributed families (Kamelin 2005; http://altaiflora.asu.ru/en/; Vaganov et al. 2019) including several species of fescues. The first person who listed species of *Festuca* in the AM was Gmelin (1747). Later, Trinius (1829) recorded six species of *Festuca* that belong to subgenera *Festuca*, *Leucopoa* (Griseb.) Hack., and *Schedonorus* Peterman. While the above mentioned authors used only macromorphological characters of flowering, and less often vegetative, shoots for species-identification, Hackel (1882) proposed using anatomical structures of the leaf blade cross-section as diagnostic characters, and these have been shown to be very significant for the taxonomy of the genus. Following Drobow (1915), botanists used these characters for the description and identification of the *Festuca* species in the Altai territory (Krylov 1914; Reverdatto 1936, 1965; Tzvelev 1976; Grubov 1982; Alexeev 1990; Chen et al. 2003; Lu et al. 2006).

In the first synopsis of the genus *Festuca* s.l. in the AM, Chusovljanov (2007) listed 22 species belonging to subgenera *Festuca* and *Leucopoa*, and described two new species for the area, namely *F.kuprijanovii* Czus. and *F.kemerovensis* Czus. (Chusovljanov 1998, 2003). *Festucakemerovensis* was recorded for the Kemerovo region, outside the AM, but Chusovljanov (2003) mentioned that the new species can be found in the Altai Mountains. To date, 21 species of *Festuca* s. l. (the fine-leaved clade and the broad-leaved clade) are reported in the Russian part of Altai (Alexeev 1990; Chusovljanov 2007; Tzvelev and Probatova 2019), 15 species in the Kazakh part of Altai (Kotukhov 2021), 9 species in the Mongolian part of Altai (Grubov 1955, 1982; Alexeev 1977, 1981; Gubanov 1996; Chusovljanov 2007; Baasanmunkh et al. 2022), and 15 species in the Chinese part of Altai (Chen et al. 2003; Lu et al. 2006). However, the taxonomic approach and treatment of *Festuca* in the AM differs, somewhat, among these countries. We can summarize that the genus Festuca s.l. comprises 22 species in the AM, including subgenus Leucopoa, sect. Leucopoa (Griseb.) Krivot. – *F.sibirica* Hack. ex Boiss.; sect. Breviaristatae Krivot. – *F.altaica* Trin., *F.tristis* Kryl. et Ivanitzk. and subgenus Festuca, sect. Festuca – *F.rubra*, *F.richardsonii* Hook., *F.borissii* Reverd., *F.kurtschumica* E. Alexeev, *F.brachyphylla* Schult. et Shult., *F.saurica* E. Alexeev, *F.lenensis*, *F.albifolia* Reverd., *F.tschujensis* Reverd., *F.pseudosulcata* Drob., *F.kemerovensis*, *F.kryloviana*, *F.pseudovina*, *F.valesiaca*, *F.oreophila* Markgr.-Dannenb., *F.rupicola* Heuff, *F.ovina* L., *F.sphagnicola* B. Keller, *F.kuprijanovii*.

Since the beginning of the XXI century, the widespread uptake of molecular sequencing methods has provided many new insights into the phylogenic relationships within many grasses (Baiakhmetov et al. 2021; Baker et al. 2021; Gallaher et al. 2022; Soreng et al. 2022). Recent phylogenetic analyses on *Festuca* s.l., based on nuclear ITS and *trnL-trnF* loci, *Festuca* is divided into two well-supported clades: narrow- or fine-leaved clade and the broad-leaved clade (Torrecilla and Catalán 2002; Torrecilla et al. 2003, 2013; Catalán et al. 2004, 2006, 2007; Inda et al. 2008; Moreno-Aguilar et al. 2022). In the AM, the fine-leaved clade is represented by the subgenus Festuca, which includes two sections: sect. Aulaxyper Dumort. (*F.rubra* L. group) and sect. Festuca (*F.ovina* group). Broad-leaved fescues comprise subgenus Drymanthele V.I. Krecz. & Bobrov, *Schedonorus* (with sections *Schedonorus* and *Plantynia* (Dumort.) Tzvel.), and subgenus Leucopoa (with sections *Breviaristatae* and *Leucopoa*). According to the latest taxonomic treatment of grasses (Soreng et al. 2022), subgenus Schedonorus is assigned to the genus *Lolium*, and subgenera *Drymanthele* (=*Drymochloa* Holub) and *Leucopoa* are regarded as independent genera. Taking all the above into account, within the genus *Festuca* in the AM, we consider here the fine-leaved fescues only.

According to the results of our molecular studies based on genome-wide genotyping (Kriuchkova et al. 2023), the Altai fescues are divided into five well-supported clades. However, relationships of some species within clades (e.g. *F.ovina*, *F.kryloviana* and *F.valesiaca*) are weakly resolved. There are also no general findings regarding the diversity of fescue species in the AM based on a comparison of the morphology and the molecular data. Therefore, a thorough study of *Festuca* in the AM, including the analysis of its morphological variability corresponding to molecular data, is necessary.

The goal of this paper is to evaluate the morphological and anatomical characteristics of particular species of fine-leaved fescues in the AM. We then present an identification key, detailed distribution maps of the examined species, and illustrations of the most important characters of the examined species.

## ﻿Materials and methods

### ﻿Study area

**Figure 1.**
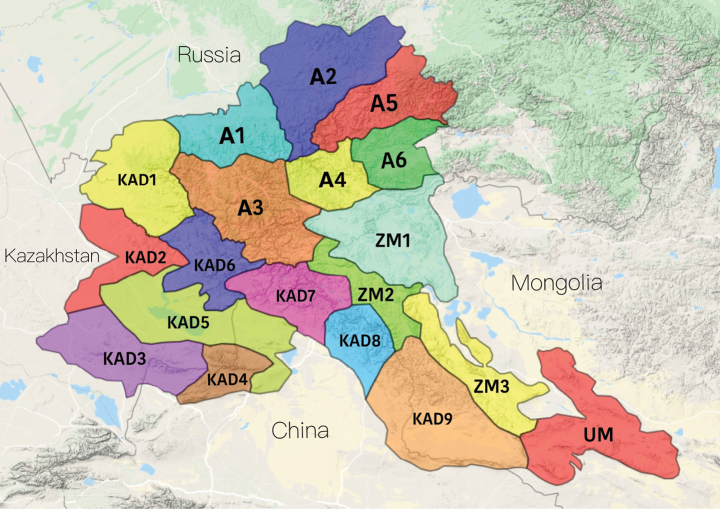
The botanical-geographical subdivision of Altai Mountains: A – Altai province (regions: A1 – Northern Altai, A2 – North-Eastern Altai, A3 – Central Altai, A4 – Tchulyshman, A5 – Abakan-Dzhebash, A6 – Khemchik); KAD – Altai-Dzungarian province (regions: KAD1 – North-Western Altai, KAD2 – Kalbinsky, KAD3 – Tarbagatai, KAD4 – Saur, KADS – Zaissan, KAD6 – Bukhtarma, KAD7 – Markakol-Kanas, KADS – Kara-Irtysh, KAD9 – Altai-Dzungarian); ZM, UM – Tuvinian-Mongolian province (regions: ZM1 – Chuya-Khobdo, ZM2 – Tsagan-Gol, ZM3 – Khobdo-Tonkhil; UM – South-Mongolian).

The study was carried out in 2017–2021 in the AM, which straddles the territories of Russia (South Siberia), eastern Kazakhstan, western China (Xinjiang Uygur Autonomous Region), and a part of western Mongolia, and occupies an area of 550,000 km^2^. The elevation varies from 240 m to the highest mountains of North Asia, namely Belukha (4506 m), and Tavan-Bogdo-Ula (4374 m). The AM include five elevational zones: steppe, forest-steppe, forest, subalpine, and alpine tundra. However, depending on the region, the composition and altitudinal boundaries of these zones are different. The AM host 2700 species of vascular plants, and the most widely distributed families are Asteraceae, Poaceae, and Fabaceae, which characterise the boreal flora (Fig. 1; Kamelin 2005; http://altaiflora.asu.ru/en/; Vaganov et al. 2019). For the AM, Kamelin (2005) proposed a botanical-geographical subdivision into regions, which we used to describe the distribution of species.

### ﻿Plant material

This study is based on the revision of the specimens deposited in the following herbaria: ALTB, AA, LE, KRA, KUZ, MW, NS, NSK, and TK. In addition, we conducted a series of field work expeditions into the Russian, Mongolian and Khazakh parts of the Altai Mountains. Herbarium acronyms follow Thiers (2023, continuously updated http://sweetgum.nybg.org/ih/). All specimens were compared with the type specimens and protologues. Photos for illustration were taken with a stereo microscope Nikon SMZ800N (Japan), the image of the general habit was taken with a scanner HP Laser Jet M1132 (USA). Maps were generated using DIVA-GIS 7.5 (Hijmans et al. 2001, continuously updated http://www.diva-gis.org). In total, more than 800 herbarium sheets of 23 species were used for the preparation of distribution maps. The distribution frequency scale is as follows: very rare – 1–10 localities, rare – 10–20 localities, common – 20–30 localities, wide spread – >50 localities.

**Figure 2.**
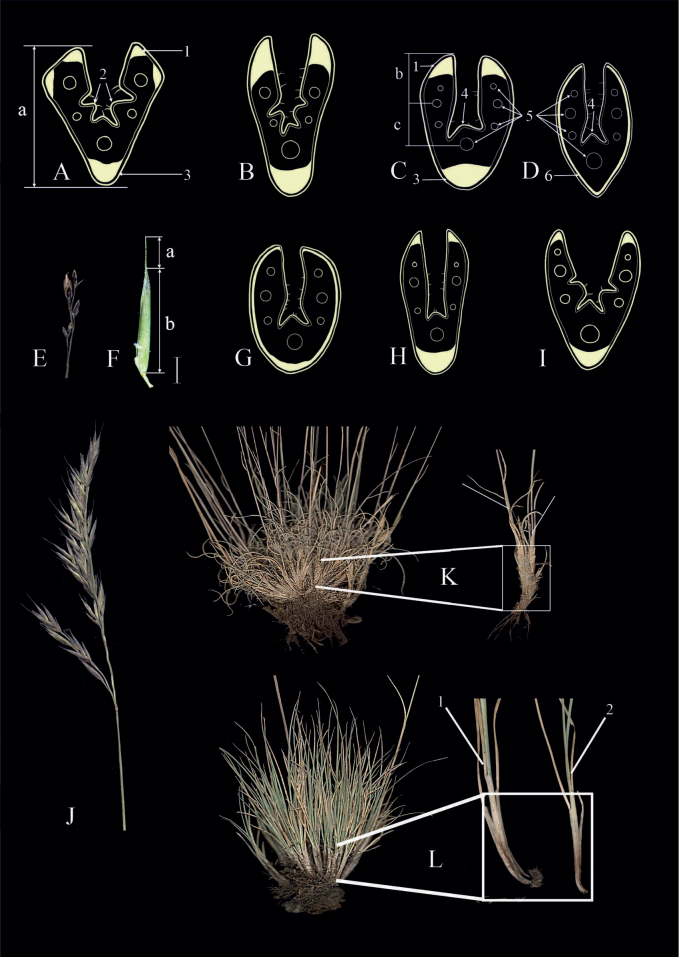
The most important macromorphological and anatomical characters of fescues **A, B, I** a cross-section with three well-defined ribs **C, D, G, H** a cross-section with only midrib or also two lateral ribs weakly defined. Shape of the cross-section leaf blades: **A** 4 angular **B, C, H** obovate **A, C, D** diagram of the leaf blades’ anatomical structure of *Festuca* (designed by E.B. Alexeev): а) diameter, b/c) ratio b/c, 1) lateral sclerenchyma strand, 2) lateral ribs, 3) keel sclerenchyma strand, 4) middle rib, 5) vascular bundles, 6) continuous sclerenchyma layer **C** lateral sclerenchyma strands similar middle strand **H** lateral sclerenchyma strands less, than middle strand **F** flower: a) awn, b) lemma **E** panicle with 1–2 spikelets on lower branches, contacted **J** panicle with more than 2 spikelets on lower branches, open **K** grouped shoots (2–3 shoots surrounded by old sheaths) **L** single shoots: 1) flowering shoot, 2) vegetative shoot (AKA tillers).

The terminology used follows Alexeev (1972, 1978, 1980, 1981, 1983, 1990), Darbyshire and Pavlick (2007), and Lu et al. (2006). The most important key characters are presented in Fig. 2.

## ﻿Taxonomic treatment

### ﻿Identification key to *Festuca* species occurring in the AM

**Table:** 

1	Anthers 0.5–1(–1.3) mm long	**2**
–	Anthers 1.4–3 mm long	**3**
2	Lower branches with more than 2 spikelets	***F.brachyphylla***
–	Lower branches with 1–2 spikelets (Fig. 2E)	***F.brevissima***
3	Sclerenchyma in leaf blade cross-section in a continuous or sometimes discontinuous layer	**4 (*F.ovina* aggr.)**
–	Sclerenchyma in leaf blade cross-section in discrete strands	**6**
4	Sclerenchyma layer thickened and wider on the midrib, invariably continuous layer (Fig. 2G); the leaf sheaths of tillers fused for ¹⁄3–½ their length	***F.kuprijanovii***
–	Sclerenchyma layer of similar width throughout, continuous or sometimes discontinuous layer (Fig. 2D); the leaf sheaths of tillers fused for ¹⁄6–¹⁄3 their length	**5**
5	Spikelets brownish	***F.sphagnicola***
–	Spikelets greenish or blueish green	***F.ovina***
6	Plants usually loosely tufted with extravaginal shoots with usually rhizomatous habit, sheaths of the tillers fused almost to their apex; shape of the leaf blade cross-section 4–6 angular (Fig. 2A)	***F.rubra***
–	Plants usually densely tufted with intravaginal shoots; shape of the leaf blade cross-section obovate to elongate (Fig. 2B)	**7**
7	Groups of 2–3 shoots are surrounded by a cover of previous sheaths (Fig. 2F)	**8**
–	Groups of 2–3 shoots are not surrounded by a cover of previous sheaths (Fig. 2L)	**10**
8	Middle sclerenchyma strand two-three times wider than lateral strands (Fig. 2H); the leaf blades flexuose	***F.tschujensis***
–	Middle sclerenchyma strand similar to lateral strand in terms of diameter; the leaf blades arcuate	**9**
9	Spikelets brownish. Endemic of Saur Mountains (KAD4)	***F.saurica***
–	Spikelets purple-green, green to white-green. Distribution in the Central and Northeast AM (А3, А5, А6)	***F.albifolia***
10	Spikelets brownish	**11**
–	Spikelets greenish	**14**
11	Sheaths of tillers fused for ²⁄3–4⁄5 their length	**12**
–	Sheaths of vegetative leaves fused for less than ²⁄3 their length	**13**
12	Sheaths of vegetative leaves fused for 4⁄5 their length; leaf blades with 7 (the narrowest ones with 5) vascular bundles, abaxial surface smooth or somewhat scabrous	***F.borissii***
–	Sheaths of vegetative leaves fused for ²⁄3–¾ their length; leaf blades with 5 (rarely 7) vascular bundles, abaxial surface distinctly scabrous	***F.kurtschumica***
13	Sheaths of vegetative leaves fused for ¹⁄3–²⁄3 their length; lemma (4.5)4.8–5.5(6) mm; leaf blades green, rare bluish-green	***F.kryloviana***
–	Sheaths of vegetative leaves usually fused for ¼–¹⁄3 their length; lemma 3.2–4.2(4.6) mm; leaf blades bluish green	***F.musbelica***
14	Leaf blade cross-section with only midrib or also two lateral ribs weak defined; sheaths of vegetative leaves fused for less than ¼–¹⁄3 their length	***F.pseudosulcata***
–	Leaf blade cross-section with 3 well-defined ribs; sheaths of vegetative leaves fused for less than ¹⁄6 their length	**15**
15	Spikelets and leaf blades bluish and covered by wax	***F.valesiaca***
–	Leaf blades green or bluish, not or rarely covered by wax	**16**
16	Spikelets 4–6 mm; lemma 2.3–4.5 mm	***F.pseudovina***
–	Spikelets 6–10 mm; lemma 4.5–6 mm	***F.rupicola***

***Festuca* L., Sp. Pl. 1: 73. 1753.**

**Type species.** —*F.ovina* L.

**Subgen. Festucasect.Aulaxyper Dumort., Observ. Gramin. Belg.: 102. 1824.**

**Type.***F.rubra* L.

***Festucarubra* L., Sp. Pl. 1: 74. 1753.**

**Type.** Lectotype (designated by Jarvis et al. 1987: 302). Habitat in Europae sterilibus siccis (GB).

**General distribution.** Widespread across North America, Eurasia, Africa, introduced in Australia and South America.

**Figure 3.**
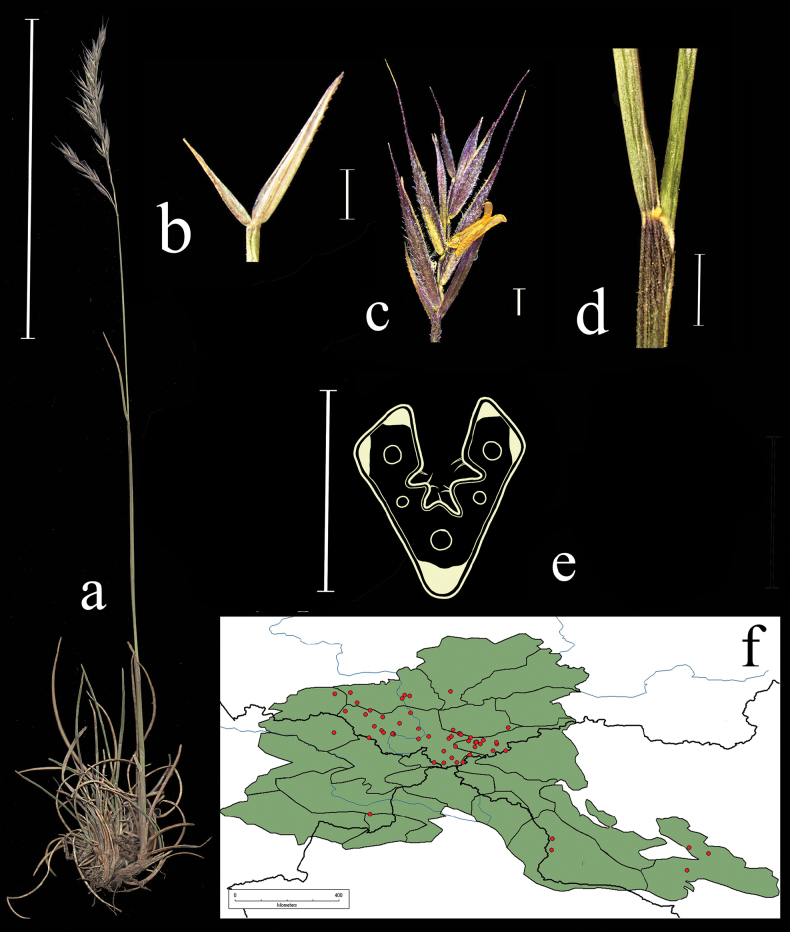
*Festucarubra***a** general habit **b** glumes, lateral view **c** spikelet, lateral view **d** junction of leaf sheath and blade, lateral view e leaf-blade cross-section f distribution map. Scale bars: 10 cm (**a**); 1 mm (**b–d**); 0.5 mm (**e**). The green colour on the map refers to information on species distribution in the region known from literature data; red points mark localities confirmed by us during the revision of herbarium materials.

**Distribution in the AM.** Widespread, all regions (Fig. 3f).

**Habitat.** Common in a wide range of vegetation types (fallow lands, roadsides, sands, meadows, forests, alpine zone); elev. 500–3500 m.

**Flowering period.** May–August.

**Chromosome number.** 2*n*=42 (Leningrad region; Alexeev et al. 1987a; Altai; Probatova and Sokolovskaya 1980; o. Vrangel; Petrovskii and Zhukova 1981; Primorskii Krai, Probatova et al. 2013); 2*n*=56 (Chukotka Peninsula; Zhukova and Petrovskii 1976; o. Vrangel; Petrovskii and Zhukova 1981).

**Notes.***Festucarubra* s.l. is an easily recognised taxon that has mostly extravaginal vegetative shoots (plants usually rhizomatous), the sheaths of the tillers and young leaves on flowering culms being fused almost to the top, 4–6 angular shape of leaf blades in cross-section, five or seven sclerenchyma strands, and well-defined ribs. *Festucarubra* is a polymorphic species: the plant length is 25–100 cm; the colour of spikelets is greenish to purplish; the surface of lemma is characterised as glabrous or smooth with hooks, prickles or pilose, and the palea keels have hooks or prickles, the number of flowers in a spikelet is 5 to 11; the number of vascular bundles in a leaf blade cross-section of vegetative shoots equals to 5–11; the lemma length is 3.7–7 mm. Recent molecular research revealed that all of the examined *F.rubra* s.l. specimens belong to one clade (Kriuchkova et al. 2023).

*Festucarichardsonii* was previously recorded for the AM. However, these specimens were misidentified with *F.rubra*. Moreover, the results of our molecular study confirmed that the above mentioned specimens did not show any significant molecular differences (Kriuchkova et al. 2023), thus we here treat the specimens collected from the AM and previously determined as *F.richardsonii* as representatives of *F.rubra*.

*Festucarichardsonii* is a species also belonging to sect. Aulaxyper. The species has a rather complicated history of records in the study area. Alexeev (1990) was the first who reported F.rubrasubsp.arctica (=*F.richardsonii*) in the AM. He noted that, in the territory of the AM, there were individuals with transitional characters between F.rubrasubsp.arctica and F.rubrasubsp.rubra. A quarter of a century later, Chusovljanov (2007) recorded three more localities of *F.richardsonii* from the AM. Generally, *F.richardsonii* is the most widely distributed grass throughout the Arctic, common throughout Eurasia, North America, and Greenland. It was also recorded in the northern boreal zones, and in the Tarbagatai, Tian Shan, and Pamiro-Alai mountains within Central Asia (Markgraf-Dannenberg 1980; Darbyshire and Pavlick 2007; Chusovljanov 2007). Typical specimens of *F.richardsonii* differ from *F.rubra* in having shorter culm length (10–30(40) cm vs 50–60 cm); shape of the panicle (contracted vs open); smaller number of spikelets on lower panicle branches (1–2 spikelets vs 2 and more spikelets); the shorter awn length (0–1.5 mm vs 1–2.3 mm), respectively.

The taxonomic history of *F.richardsonii* is challenging. In the latest taxonomic revision of the genus *Festuca* in Russia, Tzvelev and Probatova (2019) synonymised *F.cryophila*, *F.kirelowii*, F.rubrassp.arctica, F.rubrasubsp.eu-rubravar.arenariaf.arctica Hack under *F.richardsonii*. Previously, Tzvelev (1972, 1976), Alexeev (1990), Lu et al. (2006) treated *F.richardsonii*, *F.cryophila*, *F.kirelowii*, F.rubrasubsp.eu-rubravar.arenariaf.arctica Hack as synonyms of F.rubrasubsp.arctica.

*Festucarichardsonii*, F.rubrasubsp.eu-rubravar.arenariaf.arctica, *F.kirelowii* were described almost at the same time. *Festucarichardsonii* was described by Hooker (1840) from North America; F.rubrasubsp.eu-rubravar.arenariaf.arctica was described by Hackel (1882) from Arctic Europe. Festucarubrasubsp.eu-rubravar.arenariaf.arctica differs from typical *F.rubra* by the following characters: plant length 13–30 cm; dense to loose tuft; panicle with 1 to 2 spikelets on lower branches, contracted, length 20–60 mm; panicle branches hairy; lemma pubescent (data on the awn length is absent; Reverdatto 1928). *Festucakirelowii* was described by Steudel (1855) from Tarbagatai, Kazakhstan. Krechetovich and Bobrov (1934) described a new species of *F.cryophila* from Yugorsky Strait, and mentioned F.rubrasubsp.eu-rubravar.arenariaf.arctica as its synonym.

**Specimens examined.** Russia. Republic Altai, Ust-Kanskii district, village Vladimirovka 50°44'4"N, 86°22'30"E, 4 June 2020, *E.A. Kriuchkova*, *D.D. Ryzhakova*, *P.D. Gudkova* (TK; used to create Fig. 3a–e); Altai, Ust-Koksu, peschano-galechnikovyi bereg r. Katuni, 9 July 1932, *B.K. Shishkin* (LE); Altai, Oirotiya, Chuiskaya step, boloto v usti r. Elongash – na vershinah, 11 August 1937, *Akamin* (LE); Vostochnyi Altai, khr. Kurkure, oz. Sarykul, yu.v. sklon, ozernaya poima mohovo-osokovo-travyanyi ernik, 16 August 1976, *A. Galanik* (LE); Altai, Oirotiya, Koshagachskii aimak, Kuraiskii hr., ushch. r. Tobozhok, severo-zap. Sklon, krai osypi, 30 August 1937, *B.A. Shtakelberg*, *I.G. Kiorring* (LE); Altaiskii zapovednik, bereg Teletskogo ozera naprotiv sel. Yailyu, vysokotravnyi lug, 22 July 1945, *L. Tyulish* (LE); Dolina r. Vaha, srednee techenie, okolo Korolskih yurt, pesok v sosnovom boru, 17 July 1910, *V.I. Ravaelev* (LE); Altai, Oirotiya, Koshagachskii aimak, gornyi pereval iz dol. Tarhaty v dol. Usai, osokovyi kochkarnik, 27 August 1936, *A.V. Kalinina*, *L.A. Sokolova et B.K. Shishkin* (LE); Respublika Altai, Kosh-Agachskii r-on, ploskogor. Ukok, sredn. tech. r. Kalguty, lev. bereg, 49°17'N, 88°03'E, 22 July 1995, *R.V. Kamelin et al.* (KUZ); Respublika Altai, Kosh-Agachskii r-on, ploskogor. Ukok, nizhnee tech. r. Argamdzhi(srednei), 49°17'N, 87°50'E, 29 July 1998, *R.V. Kamelin et al.* (KUZ); Altaiskii krai, Charyshskii r-on, bass. r. Kumir, nizhnee tech. r. Berezovaya prav bereg, 50°53'N, 84°17'E, 3 August 1995, *R.V. Kamelin*, *et al.* (KUZ); Respublika Altai, Kosh-Agachskii r-on, dolina r. Ulandryk bliz vyhoda iz gor, 49°42'N, 89°07'E, 22 August 1998, *A.I. Shmakov*, *et al.* (ALTB); Altai, Oirotskaya avt. obl. s. Ust-Koksu, yuzhnye kamenistye sklony, 10 July 1931, *B.K. Shishkin*, *L. Chilikina* (LE); Altai, Ust-Koksinskii r-n, dolina r. Bannoi, otrogi khr. Holzun, levyi bereg reki, 8 August 1984, *M. Lomonosova*, *Bubnova* (NSK); Respublika Altai, Shebalinskii raion, v 3 km po doroge ot sela Barlyk k selu Topuchaya, levyi bereg reki Sema. Raznotravno-zlakovyi pribrezhnyi lug (*Festucapratensis*, *F.rubra*, *Dactylisglomerata*, *Elymussibiricus*, *Lathyrusvernum*) 50°27'37"N, 85°34'45"E, 3 July 2020, *E.A. Kriuchkova*, *D.D. Ryzhakova*, *P.D. Gudkova* (TK); Respublika Altai, Ust-Kanskii raion, okolo sela Vladimirovka. Kamenistyi bereg reki Charysh (*Poa* sp., *Festucarubra*, *Potentilaanserina*), 51°3'3"N, 84°12'0"E, 4 June 2020, *E.A. Kriuchkova*, *D.D. Ryzhakova*, *P.D. Gudkova* (TK); Respublika Altai, Kosh-Agachskii raion, 4 km zapadnee sela Kurai. V elnike, na beregu reki, 50°14'13.6"N, 87°50'45.2"E, 4 July 2020, *E.A. Kriuchkova*, *D.D. Ryzhakova*, *P.D. Gudkova* (TK); Respublika Altai, Kosh-Agachskii raion, 4 km zapadnee sela Kurai. Turbaza Merkit. Sredi mha, na bolotistom beregu (*Festucarubra*, *Carex* sp., *Scirpus* sp.), 50°15'15.9"N, 87°51'11"E, 6 July 2020, *E.A. Kriuchkova*, *D.D. Ryzhakova*, *P.D. Gudkova* (TK); Respublika Altai, Ongudaiskii raion, v 10 km ot sela Topuchaya na yug. Lug u dorogi 51°0'31"N, 85°38'17"E, 8 July 2020, *E.A. Kriuchkova.*, *D.D. Ryzhakova*, *P.D. Gudkova* (TK); Respublika Altai, Chemalskii raion, okrestnosti sela Chemal, pravyi bereg reki Chemalki, sklon, sosnovy les s vyhodami skal, 18 June 2017, *P.A. Kosachev*, *P.D. Gudkova* (TK); Respublika Altai, Kosh-Agachskii raion, 4 km zapadnee sela Kurai. Turbaza Merkit. Sredi mha, na bolotistom beregu (*Festucarubra*, *Carex* sp., *Scirpus* sp.), 50°15'15.9"N, 87°51'11.0"E, 6 July 2020, *E.A. Kriuchkova*, *D.D. Ryzhakova*, *P.D. Gudkova* (TK).

Kazakhstan. VKO, Ridderskii raion, bliz Riddera, Altaiskii bot. sad., boloto, 21 July 1936, *E.P. Matveeva* (LE); Semipalatinskaya obl. Semipalatinskii uezd, Chingiz, gory Mashant, gornyi lug, 8 June 1914, *C. Kossinsky* (LE).

Mongolia. Mong. Altai, Hasannu-Hairhan, sev. sklon Uagon-Irmykula, sklon sev ekspozitsii v verh. Hunkerin-ama, kobreznik, elev. 2700–3100 m, v travostoe, 23 August 1972, *V. Grubov et al.* (LE); Mong. Altai, Hasachtu-Hairhan, sev. sklon Uagan-Irmyk-ula, sklon sev. ekspozitsii v verh. Hunkerin-ama, listvennichnyi les, elev. 2500–2700 m, 23 August 1972, *V. Grubov et al.* (LE); Zaphanskii aimak, Otgon somon, yuzhn. sklon Otgon-tenger, tipchakovaya pitrogritnaya step, elev. 2450 m, Sair v doline r. Chulut, 15 July 1974, *Bagurai*, *Gambold*, *Damba*, *Muibayar* (LE); Gobi-Altai aimak, dolina r. Bidzhi-gol v Mongolskom altae v 5 km v verh po techeni. reki na predgornyi lug s reliktovymi berezami u vyhoda klyucha po sklonu ravniny, 10 August 1947, *A.A. Yunatov* (LE).

China. Sintszyan-Uigurskaya avt. obl., v. Tian-Shan, sev. sklon, bass. r. Manas, levoberezhe, dol. r. Ulan-Usu, srednyaya chast doliny, lesnoi poyas, po dnishchu doliny na galechnikah, 18 July 1957, *A.A. Yunatov*, *Li Shi-in*, *Yuan I-fen* (LE); KNR, Sintszyan-Uigurskaya avt. obl., v. Tian-Shan, severnye predgorya khr. Narat plato spuskayushchuyushcheesya v dolinu Ionma – pritok Kungesa, subalpiiskii lug, 7 August 1958, *A.A. Yunatov*, *Yuan I-Fen* (LE).

**Section Festuca.**

**Type species.***F.ovina* L.

***Festucaalbifolia* Reverd., Sist. Zametki Mater. Gerb. Krylova Tomsk. Gosud. Univ. Kuybysheva 3: 2. 1936**

≡ F.lenensissubsp.albifolia (Reverd.) Tzvel., Bot. Zhurn. 56 (9): 1254. 1971.

**Type.** Lectotype (designated by Tzvelev 1972: 39) [Russia] Khakasskii okrug, bliz ul. Birzul. Stepnoi lug na gore, 10 July 1929, *V.V. Reverdatto* (LE!).

**General distribution.** Russia (Khakassia, Western Sayan), Northern Mongolia.

**Figure 4.**
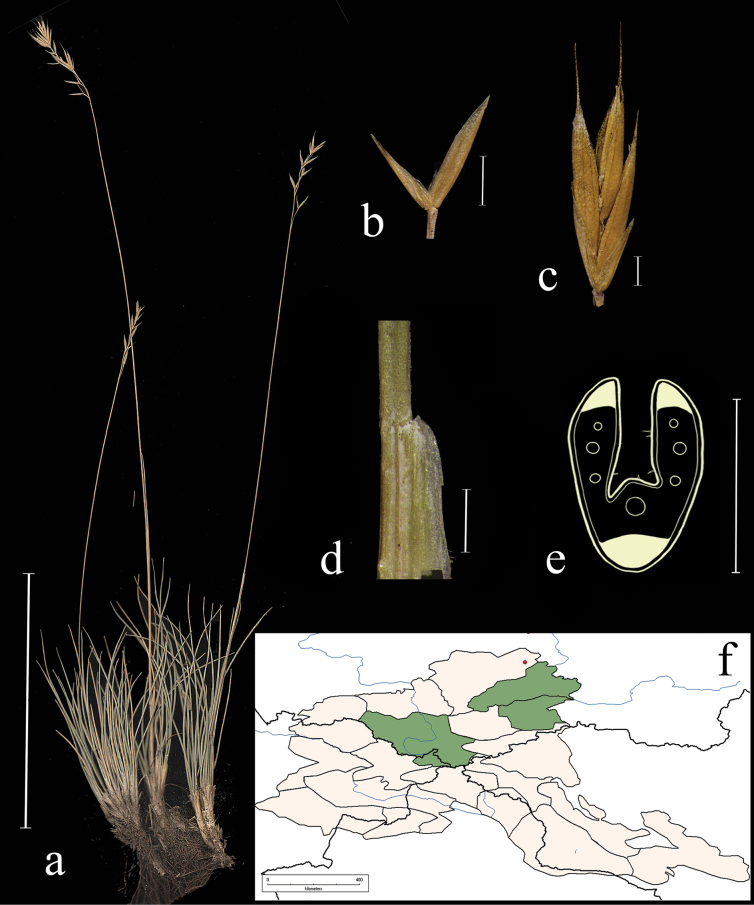
*Festucaalbifolia***a** general habit **b** glumes, lateral view **c** spikelet, lateral view **d** junction of leaf sheath and blade, lateral view **e** leaf-blade cross-section f distribution map. Scale bars: 10 cm (**a**); 1 mm (**b–d**); 0.5 mm (**e**). The green colour on the map marks information about distribution for the region known from literature data, red points mark localities confirmed by herbarium materials revised during our studies.

**Distribution in the AM.** Very rare; А3, А5, А6 (Fig. 4f).

**Habitat.** Petrophytic steppes, stony slopes, rock fissures; elev. 500–2000 m.

**Flowering period.** June–July.

**Chromosome number.** 2*n*=unknown.

**Notes.***Festucaalbifolia* was described by Reverdatto (1936) based on herbarium material from Khakassia and Altai. Previously, the species was treated as F.lenensissubsp.albifolia (Tzvelev 1971, 1972; Alexeev 1990). However, *F.albifolia* differs from *F.lenensis* by the spikelets covered with a thick layer of wax, the color of spikelets (green vs green-brown), the leaf sheaths of tillers (glabrous and smooth vs scabrous), degree of the leaf sheath fusion (fused ¼–½ their length vs fused ½ their length), ratio b/c (ca. 1 vs ca. 2).

*Festucaalbifolia* morphologically is also close to *F.valesiaca.* However, *F.albifolia* is distinguished from *F.valesiaca* by shoots (grouped by 2–3, surrounded by a cover of the old sheath vs single, not grouped), the leaf blade width (0.6–0.8 mm vs (0.35)0.4–0.6 mm), the number of ribs in a leaf blade cross-section (1 well-defined midrib vs 3 well-defined ribs), the number of vascular bundles (7 vs 5), the leaf sheaths of tillers (fused to ¼–¹⁄3 their length vs fused to ¹⁄6–¼ their length), the lemma length (4–5 mm vs (2.8)3.2–4.2(4.7) mm).

**Specimens examined.** Russia. Okr. s. Sonskogo, shchebnistii sklon. 6–8 June 1910, V. Titov; Russkii Altai. Chuiskie belki. R. Sebistei, pritok Kokuzeka. Sukhie sklony, 7 August 1911, *V. Sapozhnikov* (syntypes 2 sheaths: TK!); Altai, sev. sklon, khr. Sailyugem, uroch. Kochkor-Bas, sklon yuzhnoi ekspozitsii, 10 June 1967, *I. Yemelkin* (ALTB; used to create Fig. 4a–e; NSK); Minusinskii uezd, mezhdu oz. Shira i r. Tuimom, na shchebnistom yuzhnom sklone, 10 June 1910, *W.I. Smirnow* (LE); Khakasskii okrug, bliz ul. Birzul, stepnoi lug na gore, 10 July 1929, *V.V. Reverdatto* (LE); Oirotskaya avton. obl. Yugo-vostochnyi Altai. Chuiskaya step. V 26 km na yugo-vostok, bereg r. Irbistu, 20 July 1938, *M. Albitskaya*, *V. Eliseeva* (LE); Khakasiya, Shirinskii raion, okr. C-u ”Borec” oz. Vlasevo, kamenistaya step, 29 June 1966, *Neifeld*, *Kurochkina* (ALTB).

***Festucaborissii* Reverd., Sist. Zametki Mater. Gerb. Krylova Tomsk. Gosud. Univ. Kuybysheva 83: 8. 1965.**

**Type.** [Russia] Yuzhnyi Altai, okr. Katon-Karagaya, Narymskii khrebet ushchele Ushkungoi, alpiiskaya obl. 1 July 1920, *V.V. Sapozhnikov* (holotype and isotype TK!).

**General distribution.** Russian, Kazakh, Mongolian Altai.

**Figure 5.**
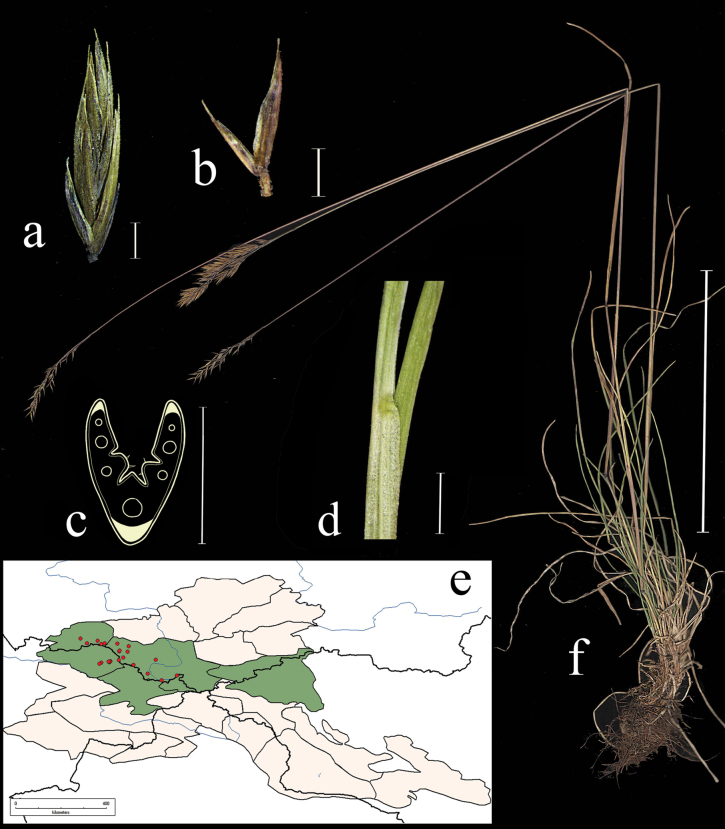
*Festucaborissii***a** spikelet, lateral view **b** glumes, lateral view **c** leaf-blade cross-section **d** junction of leaf sheath and blade, lateral view **e** distribution map **f** general habit. Scale bars: 10 cm (**f**); 1 mm (**a, b, d**); 0.5 mm (**c**). The green colour on the map refers to information on species-distribution in the region known from literature data, red points mark localities confirmed by us during revision of herbarium materials.

**Distribution in the AM.** Common; А3, ZM1, KAD1, KAD6 (Fig. 5e).

**Habitat.** Stony slopes, rock fissures, screes; thin larch and cedar-larch forests at the edge of the forest, alpine zone, riversides in mosses; elev. 500–2200 m.

**Flowering period.** July–August.

**Chromosome number.** 2*n*=unknown.

**Notes.***Festucaborissii* is close to *F.kryloviana*. Some taxonomists have distinguished the species from each other based on characters of the abaxial surface of the leaf blade and panicle branches, but these, in fact, cannot be used for this purpose (Tzvelev 1976; Alexeev 1990; Tzvelev and Probatova 2019). Both species are characterised by glabrous or scabrous leaf blades, scabrous or pubescent panicle branches. *Festucaborissii* differs from *F.kryloviana* by the structure of tillers (loosely tufted with extravaginal shoots vs densely tufted with intravaginal shoots respectively), the shape of the leaf blade cross-section (elongated elliptical vs obovate), leaf sheaths of tillers (fused for 4⁄5 their length vs fused for ¹⁄3–½ their length), the lemma length (3.5–4 mm vs 4–6 mm respectively).

**Specimens examined.** Russia. Altai. Tigiretskii kkhrebet. Vershina r. Kumir, alpiiskii lug, 5 July 1955, *A. Kuminova*, *M. Mitrofanova* (ALTB; used to create Fig. 5a–d, f); Zapadnyi Altai, Kkhrebet Ivanovskii 2.5 km sev-vost. versh. Vysheivanovskii Belok, na skalah, elev. 2100 m, 18 July 1997, *D.V. Chusovljanov* (KUZ); Altaiskii krai, Zmeinogorskii r-n, kkhrebet Tigiretskii belok, 51°03'N, 82°56'E, 22 July 1997, *A.N. Kupriyanov* (KUZ); Tomskaya gub., Zmeinogorsk. u., kamen. sklony, subalp. obl., g. Sinyuha, bliz Ridderskogo rud., 19 June 1909, *V.S. Ilin* (LE); Tomskaya gub., Biiskii uezd: Katunskie belki, dolina r. Prohodnoi, lstvennichnyi les, 1 June 1911, *W.L. Nekrassowa* (LE); Altai, khr. Korgon verh. r. Tatarki, alpiiskii lug, 4 July 1953, *A. Kuminova*, *G. Pavlova* (LE); Tomskaya gub. Biiskii u. Katunskie belki, verhove Katuni, vodorazdel s Bel. Berelyu, alpiiskie luga i tundra, 7 July 1911, *W.L. Nekrassowa* (LE); Tomskaya gub. Zmeinogorsk. u. dolina r. Bystruhi, po sklonu k gornomu ruchyu bliz lesnogo predela, 2 August 1910, *P. Tomin* (LE); Altai, khr. Holzun, istoki r. Bannoi, alpiiskii lug, 8 August 1984, *N. Frizen* (NSK); Altai, Ust-Koksinskii r-n, Terektinskii hr., istoki r. Kastahta, osokovo-raznotravnyi kobrezev lug, 12 August 1984, *Frizen*, *Bubnova* (NSK); Altai, khr. Korgon, verhovya r. Charysha, alpiiskii lug, 2 June 1953, *A. Kuminova*, *G. Pavlova* (LE).

Kazakhstan. Vostochnyi Kazakhstan, Ivanovskii hr., okr. Leninogorska, kurumnik, razrezhennyi listvenichnik, 10 September 2001, *D. Chusovljanov* (KUZ); Vostochnyi Kazakhstan, Ivanovskii hr., okr. Leninogorska, sypuha, 10 September 2001, *D.V. Chusovljanov* (KUZ); Vostochno-Kazakhstanskaya oblast, Khoazunskii kkhrebet pravyi bereg pravogo pritoka r. Khaidun na lugu na shchebnistoi pochve, 22 June 1939, *Paulskaya* (LE); Vostochnyi Kazakhstan, Koksinskii hr., elev. 1800 m, nizkotravnye alpiiskie luga, 10 August 2004, *Yu. Kotuhov.* (KUZ); Zapadnyi Altai, Khrebet Ivanovskii, v raione ozer Beloubinskih, elev. 1600 m, razrezhennyi kedrach, 18 July 1976, *Yu. Kotuhov* (KUZ); Zapadnyi Altai, Khrebet Ivanovskii, yugo-vostochnye otrogi versh. Vysheivanovskii Belok. Goltsy. elev. 2300 m, 26 July 1997, *D.V. Chusovljanov* (KUZ); Zapadnyi Altai, kh. Ivanovskii, Vostochnoe podnozhie versh, Vysheivanovskii Belok, shchebnistaya tundra, 26 July 1997, *D.V. Chusovljanov* (KUZ); Zapadnyi Altai, Khrebet Ivanovskii 3.5 km sev-vost. versh. Vysheivanovskii Belok, alpiiskii lug, elev. 1900 m, 8 July 1997, *D.V. Chusovljanov* (KUZ).

***Festucabrachyphylla* Schult & Schult fil., Mant. 3: 646. 1827.**

≡ F.ovinasubsp.brevifolia (R. Br.) Hack., Monogr. Festuc. Europ.: 117. 1882.

**Type.** Lectotype (designated by Frederiksen 1977: 269) Melville Island, 1820, *Mr. Edwards* (BM; syntypes BM, LE!).

**General distribution.** Common throughout all arctic parts of NE European Russia, Siberia, Beringia, Canada, and Greenland. It also occurs in the northern boreal zones and in mountains farther south of Central Asia.

**Figure 6.**
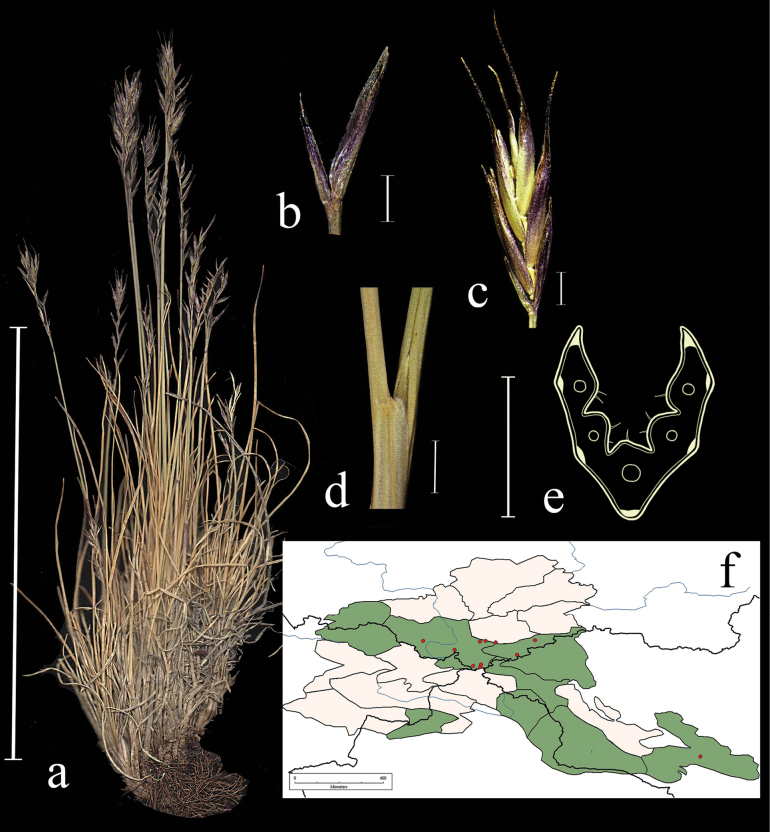
*Festucabrachyphylla***a** general habit **b** glumes, lateral view **c** spikelet, lateral view **d** junction of leaf sheath and blade, lateral view **e** leaf-blade cross-section **f** distribution map. Scale bars: 10 cm (**a**); 1 mm (**b–d**); 0.5 mm (**e**). The green colour on the map refers to information on species-distribution in the region known from literature data, while red points mark localities confirmed by us during revision of herbarium materials.

**Distribution in the AM.** Rare; KAD1, KAD4, KAD8, KAD9, А3, ZM1, ZM2, UM (Fig. 6f).

**Habitat.** Mountain steppes, alpine zone, rocky slopes, rocks and pebbles, screes; elev. (2000)2500–3500 m.

**Flowering period.** July–August.

**Chromosome number.** 2*n*=42 (Kamchatskaya obl., Magadanskii obl.; Alexeev et al. 1987b; o. Vrangelya; Zhukova and Petrovskii 1980; Chukotka; Yurtzev and Zhukova 1978; American Arctica; Frederiksen 1981)

**Notes.***Festucabrachyphylla* is easily recognised by its panicles with more than 2 spikelets on lower branches, anthers 0.5–1.5 mm in length, scabrid leaf blades, 5 or 7 sclerenchyma strands. Phylogenetically, our molecular study demonstrated that *F.brachyphylla* and *F.brevissima* refer to a separate clade distinct from sect. Festuca (Kriuchkova et al. 2023).

**Specimens examined.** Russia. Kuraiskii khrebet, yuzhnyi makrosklon, alpiiskaya luzhaika, na osypyah. elev. 2850 m, 12 July 1998, *D.V. Chusovljanov* (KUZ; used to create Fig. 6a–e); Respublika Altai, Kuraiskii hr., verhove r. Ortalyk, po beregu ruchya, 2 July 1999, *A.A. Ebel* (KUZ); Altai, Oirotskaya avt. obl. r. Archaly, pritok r. Koksu, alp. mohovo-lishainikovaya tundra, 27 July 1931, *B. Shishkin*, *L. Chilinina*, *G. Sumnevich* (LE); Altai, Oirotskaya avt. obl. pereval mezhdu Ak-kemom i Kairom, alpiiskie luga, 18 July 1931, *B. Shishkin*, *L. Chilinina*, *G. Sumnevich* (LE); Altai, Oirotskaya avt. obl., istoki r. Kanasa, alpiiskaya tundra, 3 August 1931, *B.K. Shishkin*, *L. Chilinina*, *G. Sumnevich* (LE); Respublika Tuva, Mugur-Aksynskii r-n, kotl. oz. Hindiktig-Hol, okr. oz. Durug-Bazhi-Kara-Hol, 50°19'N, 89°55'E, 5 July 1995, *R.V. Kamelin* (ALTB); Altai, Kosh-Agachskii aimak, okr. s. Tashanta, bereg ozera, 15 July 1958, *A. Kuminova* (TK); Gorno-Altaiskaya avt. obl. Ulaganskii r-n, verhove r. Yarly Amry, elev. 2700 m, 50°15'N, 87°40'E, driadovaya tundra, 6 August 1981, *M. Danilov*, *N. Kolesnikova* (NS); Kuraiskii khr. yuzhnyi makrosklon, v verhove Ortolyk, alpiiskii lug, na osypi, elev. 2850 m, 12 July 1998, *D.V. Chusovljanov* (KUZ); Respublika Altai, Kosh-Agachskii raion, plato Ukok, pereval Teplyi klyuch, elev. 2900 m, 49°24'27"N, 88°02'09"E, 7 August 2001, *A.I. Shmakov*; Respublika Altai, Kosh-Agachskii raion, khr. Sailyugem, okr. Oz. Karakul, 49°40'N, 88°32'E, 3 July 2001, *M.G. Kucev*, *D.A. German* (ALTB).

Mongolia. Istoki r. Humet, kamenistaya tundra, 10 August 1998, *D.V. Chusovljanov* (KUZ); Kobdoskiy aymak, khr. Munkh-Khayrkhan, bassein r. Ulystyn-gol bliz severnoi chasti lednika Munkh-Khayrkhan, elev.3250 m 12.08.1991, G. N. Ogureeva (MW).

***Festucabrevissima* Jurtzev, Bot. Zhurn. 57 (6): 645. 1972.**

**Type.** [Russia] Zap. Chukotka, istoki Anadyrya, u yuzhn. berega oz. Edgygytgyn, na yugo-zap. sklone, 20 July 1968, *B. Yurtzev et al. n K-61* (holotype LE!).

**General distribution.** Arctic parts of Siberia, Beringia, Altai.

**Figure 7.**
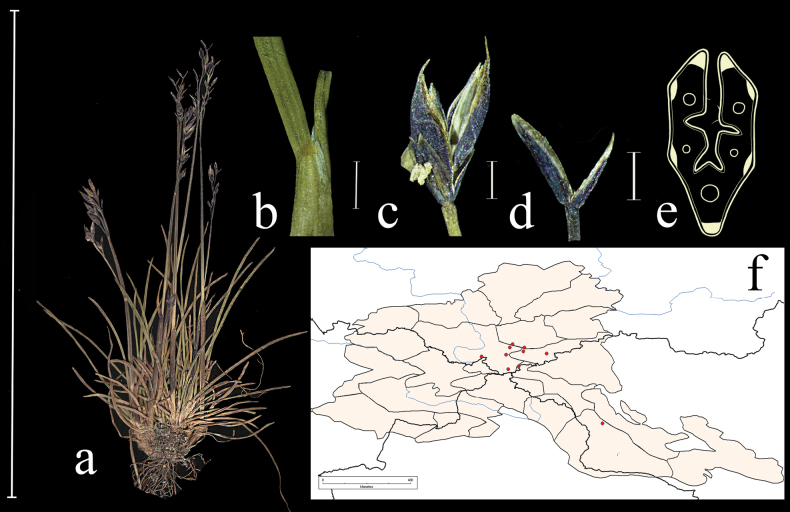
*Festucabrevissima***a** general habit **b** junction of leaf sheath and blade, lateral view **c** spikelet, lateral view **d** glumes, lateral view **e** leaf-blade cross-section **f** distribution map. Scale bars: 10 cm (**a**); 1 mm (**b–d**); 0.5 mm (**e**).

**Distribution in the AM.** Very rare; A3, A4, ZM1 (Fig. 7f).

**Habitat.** Alpine zone, screes; elev. 2000–3500 m.

**Flowering period.** July–August.

**Chromosome number.** 2*n*=14 (Russia, Chukotka; Yurtzev and Zhukova 1978; Kamchatka; Probatova et al. 2008).

**Notes.***Festucabrevissima* is distributed in Chukotka, Kamchatka, Wrangel Island and Alaska on gravel slopes. As a result of the herbarium revision, we recorded the species much farther to the South in the mountains of Siberia. The specimens have been misidentified as *F.brachyphylla*. *Festucabrevissima* differs from *F.brachyphylla* by the number of spikelets in the panicle (fewer than 8 spikelets vs more than 11 spikelets respectively), the number of spikelets on lower branches (1–2 spikelets vs 2- or more spikelets), the panicle length (0.7–26 mm vs 23–55 mm), the lemma length (2.5–4 mm vs 4.5–5.5 mm), the plant length (up to 120 mm vs 100–550 mm). According to the results of our molecular studies, *F.brevissima* is clearly separated from *F.brachyphylla* (Kriuchkova et al. 2023). All the localities listed above are new records of the species for the AM. Information on the occurrence of species in areas previously unnoticed or misidentified with other species is nowadays very important, because being under increasing pressure from human activities and recently also under the negative impact of global climate warming, the dynamics of both flora and vegetation in recent decades is much faster than before (Araújo and Rahbek 2006; Bellard et al. 2012; Vintsek et al. 2022; Nobis et al. 2023).

**Specimens examined (New records)**: Russia. Gorno-Altaiskaya A.O., Kosh-Agachskii r-n, urochishche Tueryk, elev. 2900 m, 50°5'N, 88°22'E, osypi kristallicheskikh slantsev, 30 June 1982, *M. Danilov*, *V. Doronkin* (LE, NS); Gorno-Altaiskaya A.O., Ulaganskii r-n, okr. oz. Choibekkol. elev. 2850 m, 50°16'N, 87°25'E, skaly po grebnyu khrebta, 13 July 1982, *M. Danilov*, *A. Grinev* (LE); Respublika Altai, Kosh-Agachskii raion, Yuzhno-Chuiskii khr., verkhovya r. Taldura bliz lednika, elev. 2480 m, 49°51'N, 87°43'E, 13 July 1993, *R.V. Kamelin et al.* (ALTB); Talduair, alp. poyas, elev. 3000 m, Kobrezievaya pustosh, 10 July 1999, *A. Ebel* (KUZ); Yuzh. makroskl. Kuraiskogo khr. v verkh. Ortolyk, alpiiskaya luzhaika, na osypi, elev. 2850 m, 12 July 1999, *A. Ebel* (KUZ); Gornii Altai, Katunskii kkhrebet, oz. Akkem, severnii sklon starogo kara, osyp, elev. 2400 m, 3 July 1974, *N.V. Revyakina*, *N. Vorobeva* (NS); Respublika Altai, Kosh–Agachskii raion, khr. Tabyn-Bogdo-Ula, ushchele Kara Chad, verkhovya reki, 49°19'N, 87°42'E, sklony ushchelya, skaly, 13 July 1992, *R.V. Kamelin*, *A. Shmakov*, *P. Golyakov*, *M. Mikhailova*, *S. Dyachenko*, *A. Kiselev*, *T. Krestovskaya*, *M. Kashcheev* (ALTB); Altai Republic, Koch-Agachskii distr., Ukok Plateau, pass between the rivers Djumala and Usay, elev. 2680 m, 49°25'35"N, 88°07'58"E, 20 July 1999, *A.I. Shmakov*, *S.V. Smirnov*, *E.V. Antonyuk*, *S.A. Kostjukov*, *V.I. Dorofeyev*, *I.N. Chubarov*, *P.A. Kosachev*, *S.A. Djachenko* (ALTB; used to create Fig. 7a–e).

Mongolia. Kobdoskiy aymak, khr. Munkh-Khayrkhan, bassein r. Ulystyn-gol bliz severnoi chasti lednika Munkh-Khayrkhan, elev. 3250 m, 12.08.1991, G. N. Ogureeva (MW).

***Festucakryloviana* Reverd., Sist. Zametki Mater. Gerb. Krylova Tomsk. Gosud. Univ. Kuybysheva 2: 3. 1927.**

**Type. *Lectotype*** (designated by Tzvelev 1972: 40) [Russia] Gorno-Alt. u., kkhrebet ot r. Berezovki do r. Khaisyna, alpiiskii lug, 1 July 1920, *V. Sapozhnikov* (LE01011329!).

**General distribution.** China (Altai, Dzungaria), Mongolia (Altai) and Russia (Altai, Sayan, Ural, Tarbagatai), Kazakhstan (Altai, Tyan-Shan,).

**Figure 8.**
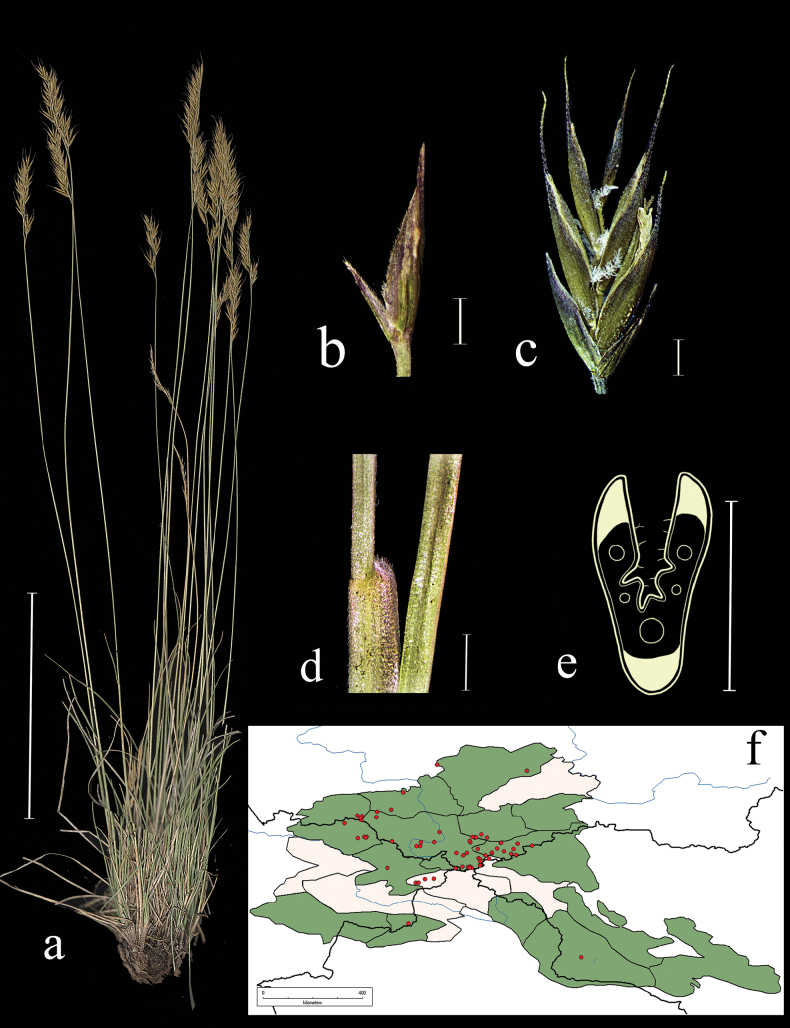
*Festucakryloviana***a** general habit **b** glumes, lateral view **c** spikelet, lateral view **d** junction of leaf sheath and blade, lateral view **e** leaf-blade cross-section **f** distribution map. Scale bars: 10 cm (**a**); 1 mm (**b–d**); 0.5 mm (**e**). The green colour on the map refers to information on species-distribution in the region known from literature data, red points mark localities confirmed by us during revision of herbarium materials.

**Distribution in the AM.** Widespread; А1, А2, А3, А4, А6, ZM1, ZM3, KAD1, KAD3, KAD4, KAD6, KAD8, KAD9, UM (Fig. 8f).

**Habitat.** Forests, stony slopes, alpine and subalpine zone, petrophytic steppes, screes, riverbanks; elev. 1000–3500 m.

**Flowering period.** July–August.

**Chromosome number.** 2*n*=28, 42 (Russia, Altai; Probatova and Sokolovskaya 1980); 2*n*=42 (Irkutsk region; Chepinoga et al. 2010).

**Notes.***Festucakryloviana* is a morphologically variable species. The surface of lemma can be glabrous and smooth or scabrous, abaxial surface of the vegetative leaf blade is glabrous to scabrous, adaxial surface of the vegetative leaf blade is covered by hooks or prickles, rarely with microhairs present, the number of vascular bundles is 5 to 7, rarely 9 and the number of ribs is 3 to rarely 5, the lemma length equals to 4.3–5 mm, the awn length varies from 2 to 5 mm; the wax on the leaf blades is present or absent.

**Specimens examined.** Russia. Respublika Altai, Kosh-Agachskii raion, srednee techenie reki Ortolyk, levoberezhe, listvennichnik, elev. 2100 m, 50°17'33.1"N, 87°50'53.8"E, 5 July 2020, *E.A. Kriuchkova*, *D.D. Ryzhakova*, *P.D. Gudkova* (TK; used to create Fig. 8a–e). Yuzhnyi makrosklon Kuraiskogo hrebta, alpiiskaya luzhaika na osypyah, elev. 2850 m, 12 July 1999, *A.A. Ebel* (KUZ); Altai, Kosh-Agachskii r-n, khr. Sailyugem, verh. r. ZHumaly, elev. 2600 m, okr. rudnika Nov. Kalguty. Nadpoimennaya terrasa, raznotravno-lukovaya alpiiskaya luzhaika v ponizhenii, 51°N, 89°E, 8 August 1982, *V. Hanminchun*, *N. Frizen*, *V. Petrusenko* (NSK); Altaiskii krai, Soloneshenskii r-n, verhovya r. Shepeta, berezkovo-lishainikovaya tundra, 51°16'N, 84°15'E, 25 June 1997, *T.O. Strelnikova*, *D.A. German* (ALTB); Altai, Kosh-Agachskii r-n, khr. Sailyugem, verh. r. ZHumaly, elev. 2600 m, okr. rudnika Nov. Kalguty. Nadpoimennaya terrasa, osochkovo-ovsyanitsevaya tundra, 51°N, 89°E, 8 August 1982, *V. Hanminchun*, *N. Frizen*, *V. Petrusenko* (NSK); Respublika Altai, Kuraiskii khr. reka Ortolyk, zakustarennyi sklon, 2 July 1999, *A.A. Ebel* (KUZ); Altai, Oirotiya, Koshagachskii aimak, Kuraiskii khr., ushchele r. Tobozhok, yugo-zap. travyanistyi sklon ushchelya Dzhayat, 3 August 1937, *Shtakelberg* (LE); Tomskaya gub. Biiskii u. belok mezhdu r. Ini i vershinoi Senteleka v 15 km ot d. Pokrovka, 16 July 1913, *N.J. Kusnezow* (LE); Altai, Oirotiya, Koshagachskii aimak, dol. r. Tarhatty, lev. ber. sklon sev-vost. ekspozitsii, 23 August 1936, *A.V. Kalinina*, *L.A. Sokolova*, *B.K. Shishkin* (LE); Altai, Tigirekskii khrebet, vershina r. Belogolosov, Korgon, vysokogornaya tundra, 12 June 1955, *A. Kuminova*, *G. Pavlova* (LE); Altai, Kosh-Agachskii aimak, yuzhn. sklon Kuraiskogo hrebta, alpiiskaya tundra, 23 July 1955, *A. Yakubova*, *E. Tyurina*, *L. Zubkus* (LE); Altai, Oirotskaya avt. obl. istoki r. Kanasa, na lednikovyh morenah, 1 August 1931, *B. Shishkin*, *L. Chilikina*, *G. Sumnevich* (LE); Centr Altai, khr. Holzun, istoki r. Bannoi, alp. poyas, na shchebnistom lugu, 8 August 1984, *L. Malyshev* (NSK); Khakasiya, Tashtynskii r-n, okr. p. Nizhnie Siry, r. Tashtyi, ostepnennyi sklon, 29 June 1983, *Bondareva* (NSK).

Kazakhstan. Yuzhnyi Altai, khr. Azutau, okresnosti s. Urunhaiki, vostochnyi sklon, razrezhennyi listvennichnyi les, 24 July 1984, *Yu. Kotuhov* (KUZ); Vostochno-Kazakhstanskaya oblast, Zapadnyi Altai, khrebet Ivanovskii, severo-vostochnyi sklon vershiny Vysheivanovskii Belok, shchebnistaya tundra, elev. 2150 m, 22 July 1997, *D.V. Chusovljanov* (KUZ); Zapadnyi Altai, khrebet Ivanovskii, yugo-vostochnye otrogi versh. Vysheivanovskii Belok, goltsy, elev. 2300 m, 26 July 1997, *D.V. Chusovljanov* (KUZ); Yugo-Zapadnyi Altai, VKO, Kolyvanskii hr., vershina sklona v verhovyah Tigireka, 25 July 1939, *Koroitkevich* (LE); Ozero Markakol, gornye luga, vysoko nad ozerom, 7 August 1908, *B.A. Keller* (LE); Khr. Saur, subalpiiskii lug, elev. 1900 m, vodorazdel r.r. B. i Mal. Dusemeneya, 5 July 1931, *N. Goncharov*, *P. Borisova* (LE).

Mongolia. Vostochnaya chast Mongolskogo Altaya, dolina r. Urhugol, listvenichnyi les po vostochnomu sklonu gory, 19 August 1930, *E.G. Pobedimova* (LE).

***Festucakurtschumica* E. Alexeev, Novosti Sist. Vyssh. Rast. 13: 24. 1976.**

**Type.** Vostochnii Kazakhstan, Altai, gory u severnogo berega ozero Markakol, vyshe granitsy lesa, 2400–2700 m, 10 July 1912, *A. Sedelnikov* (holotype LE!).

**General distribution.** Mongolia (Altai), Kazakhstan (Altai), Russia (Altai, Olkhon island, lake Baikal).

**Figure 9.**
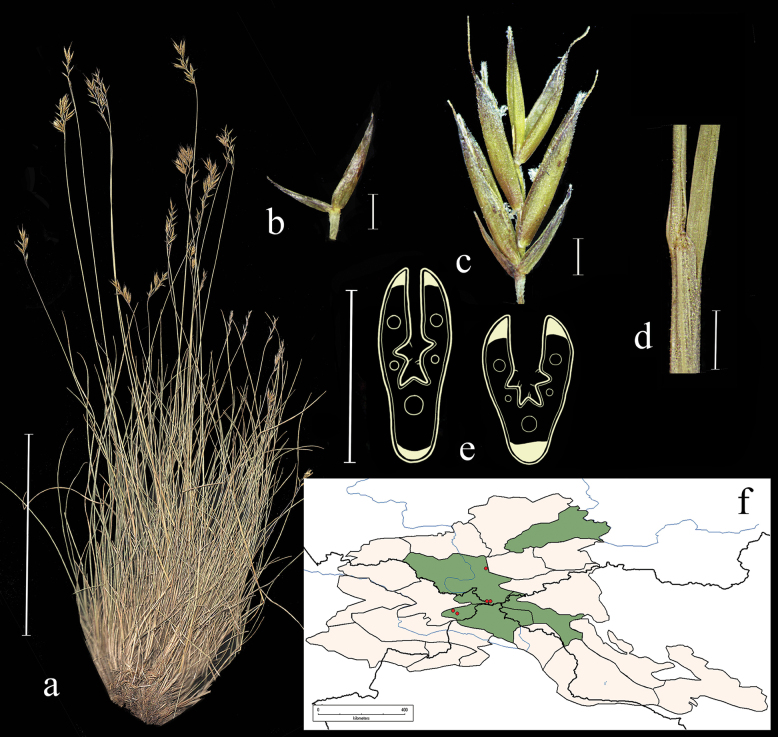
*Festucakurtschumica***a** general habit **b** glumes, lateral view **c** spikelet, lateral view **d** junction of leaf sheath and blade, lateral view **e** leaf-blade cross-section **f** distribution map. Scale bars: 10 cm (**a**); 1 mm (**b–d**); 0.5 mm (**e**). The green colour on the map refers to information on species-distribution in the region known from literature data, red points mark localities confirmed by us during revision of herbarium materials.

**Distribution in the AM.** Very rare; А3, А5, ZM2, KAD7 (Fig. 9f).

**Habitat.** Gravelly places, screes; elev. 1500–3500 m.

**Flowering period.** July.

**Chromosome number.** 2*n*=unknown.

**Notes.** The distribution of *Festucakurtschumica* is restricted to the AM. *Festucakurtschumica* is similar to *F.kryloviana* but differs by leaf sheaths of tillers (fused for ²⁄3–¾ their length vs fused for ¹⁄3–½ their length), leaf blade width (0.4–0.5 (0.55) mm vs (0.4)0.55–0.85(1) mm), lemma length (3.5–4.5 mm vs 4.5–6 mm), and the number of vascular bundles (5 vs (5)7 respectively). However, in accordance with the results of molecular analysis, *F.kurtschumica* and *F.kryloviana* are grouped into a common clade (Kriuchkova et al. 2023). The species needs a taxonomic revision within its entire distributional range, including the locus classicus, namely the vicinity of Lake Markakol, the Khazakh part of the AM.

**Specimens examined.** Russia. Altai, Oirotskaya avt. Obl., r. Archaly, pritok r. Koksu, alpiiskaya mokhovo-lishainikrvaya tundra, 27 July 1931, *B. Shishkin*, *L. Chilikina*, *G. Sumnevich* (paratype LE!); Respublika Altai, Kosh-Agachskii r-on, zap. chast ploskogor. Ukok, vostochnyi makrosklon gory Muzdy-Bulak, 49°15'N, 87°14'E, 23 July 1998, *R.V. Kamelin*, *A.I. Shmakov*, *S. Kostyukov*, *I. Chubarov*, *D. Tihonov*, *E. Antonyuk* (ALTB); Respublika Altai, Kosh-Agachskii r-on, ploskogor. Ukok, okresnosti oz. Ukok, 49°15'N, 87°23'E, 26 July 1998, *R.V. Kamelin*, *A.I. Shmakov*, *S. Smirnov*, *P. Kosachev*, *D. Tihonov*, *E. Antonyuk* (KUZ); Altai, Oirotskaya avt., obl. r. Archaly, pritok r. Koksu, alp. mohovo-lishainikovaya tundra, 27 July 1931, *B.K. Shishkin*, *L. Chilinina*, *G. Sumnevich* (LE); Respublika Altai, Ulaganskii raion, Kuraiskii hr., verhovya r. Yarly-Amry, elev. 2700–2900 m, 50°20'08"N, 87°44'45"E, 20 July 2012, *A.I. Shmakov et al.* (ALTB).

Kazakhstan. Yuzhnyi Altai, khr. Azutau, Urunhaiskii pereval, razrezhennyi listvennichnyi les, ostepnennye lugoviny, elev. 1800 m, 20 June 1986, Yu. Kotuhov (ALTB; used to create Fig. 9a–e); Yuzhnyi Altai, khr. Azutau, Urunhaiskii pereval, elev. 1800 m, razrezhennyi listvennichnyi les, ostepnennye lugoviny, 20 June 1986, *Yu. Kotuhov* (KUZ); Vostochnyi Kazakhstan, Altai, gory u severnogo berega oz. Markakol vyshe granitsy lesa, elev. 2400–2700 m, 10 July 1912, *A. Sedelnikov* (LE).

Mongolia. Sev. Mongolia i Hangai, listvennichnyi les, v dvuh verstah ot Klyucha Hurum-bulyk, 8 July 1926, *N. Ikonnikov-Galitzky* (LE); Sev. Mongolia i Hangai, okr. TSzain-shaby, v listvennichnom lesu, k zapadu ot klyucha Haltszangyn-bulyk, 17 July 1926, *J. Prochanov* (LE); Zapadnaya Mongoliya, Bayan-Ulgiiskii aimak. Mongolskiy Altay, 15 km na zapad ot Khoto-Nura, subalpy na granitse s Kitayem, 17 August 1979, *I.A. Gubanov*, Det. Alexeev E.B. (MW0170759); Zapadnaya Mongoliya, Kobdoskii aimak, Mongolskii Altai. Vostochnyi makrosklon khr. Munkh-Khayrkhan, kotlovina Ikh-Khak v verkhovyakh r. Dolon-Nuryn-gol bliz yuzhnyi chasti lednika Munkh-Khayrkhan, vykhody slantsev na verkhushke gory, elev. 3020 m, 7 August 1991, *G.N. Ogureeva*, Det. Dariyma (MW0170755).

***Festucamusbelica* (Reverd.) Iconn. Opred. Vyssh. Rast. Badahsh.: 75. 1979.**

≡ F.krylovianavar.musbelica Reverd., Sist. Zametki Mater. Gerb. Krylova Tomsk. Gosud. Univ. Kuybysheva 2: 4. 1927.

= F.ovinasubsp.sulcatavar.hypsophila St.-Yves., Candollea 111. 1932; non *F.hypsophila* Phil. Anal. Mus. nac. Chile: 89. 1891.

= *F.oreophila* Markgr.-Dannenb., Willdenowia 11: 208. 1981.

**Type. *Lectotype*** (designated by Tzvelev 1972: 168) Armenia, distr. Nor. – Bajazet, in monte Achdagh mjor, 16 August 1929, *O. Zedelmejer*, *T.Heidemann* (F.ovinasubsp.sulcatavar.hypsophila; LE!).

**General distribution.** The species occurs in mountains, from Turkey, throughout Caucasus, Central Asia (Western, Eastern Siberia), up to Mongolia (Altai).

**Figure 10.**
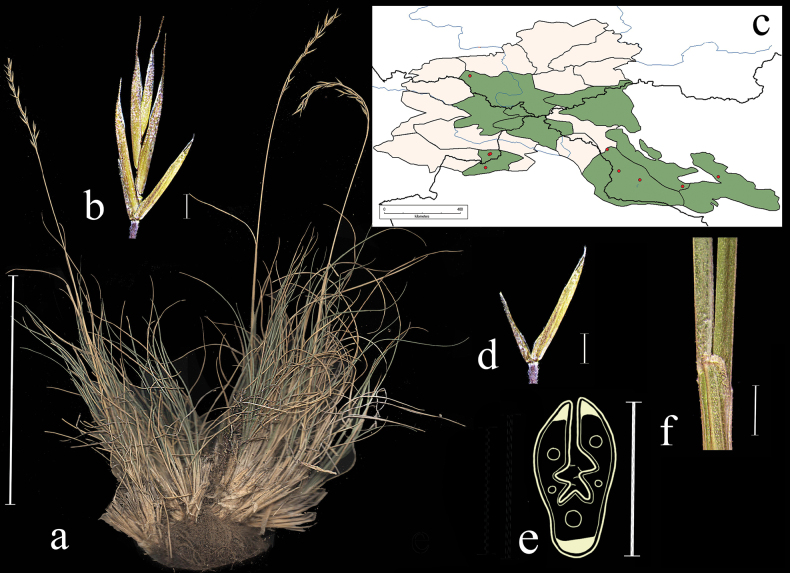
*Festucamusbelica***a** general habit **b** spikelet, lateral view **c** distribution map **d** glumes, lateral view **e** leaf-blade cross-section **f** junction of leaf sheath and blade, lateral view. Scale bars: 10 cm (**a**); 1 mm (**b, d, f**); 0.5 mm (**e**). The green colour on the map refers to information on species-distribution in the region known from literature data, red points mark localities confirmed by us during revision of herbarium materials.

**Distribution in the AM.** Very rare; А3, ZM1, ZM3, KAD4, KAD6, KAD9, UM (Fig. 10c).

**Habitat.** Alpine zone, forests, meadows, petrophytic steppes; elev. 1500–2500 m.

**Flowering period.** July–August.

**Chromosome number.** 2*n*=14 (Kazakhstan, Kyrgyzstan; Alexeev et al. 1988; TRANS-Baikal territory; Probatova et al. 2013).

**Notes.**Festucakrylovianavar.musbelica was described from the Altai Republic, Russia (Reverdatto 1927), whereas F.ovinasubsp.sulcatavar.hypsophila was described from Europe (Saint-Yves 1932). Tzvelev (1971), in his taxonomic revision of the genus, proposed new nomenclatural combinations: F.valesiacasubsp.musbelica and F.valesiacasubsp.hypsophila and respectively referred F.krylovianavar.musbelica and F.ovinasubsp.sulcatavar.hypsophila to the synonyms of the above-mentioned taxa. Festucakrylovianavar.musbelica is rather well distinguished from F.krylovianavar.kryloviana by the characters of the leaf sheath surface of tillers (glabrous or smooth and glossy vs scabrous and matte), the leaf sheaths of tillers (fused for ¼ their length, rarely ¹⁄6 their length vs fused for ¹⁄3–½ their length). Considering these major differences, Ikonnikov (1979) proposed to raise F.krylovianavar.musbelica to the rank of species, *F.musbelica*. Later, Markgraf-Dannenberg (1981) described a new species, *F.oreophila*, and synonymized F.ovinasubsp.sulcatavar.hypsophila under this new neme. In the later taxonomic treatment of the genus *Festuca*, Tzvelev and Probatova (2019) subsumed F.ovinasubsp.sulcatavar.hypsophila, F.krylovianavar.musbelica, F.valesiacasubsp.hypsophila, and *F.oreophila* as synonyms of *F.musbelica. Festucamusbelica* is the earliest legitimate name and thus, according to ICBN, chapter 2, article 11.4 (Turland et al. 2018), has priority as the correct name.

Molecular analyses placed *F.musbelica* in the common clade with *F.valesiaca* (Kriuchkova et al. 2023). However, the species differs from *F.valesiaca* by the color of spikelets (brown vs green, bluish green, pinkish green), and the leaf sheath of tillers (fused for ¼–¹⁄3 their length vs fused for ¼–¹⁄6 their length). The species needs a taxonomic revision over its entire distributional range.

**Specimens examined.** Russia. Gornyi Altai, Ust-Kanskii r-n, lev. bereg r. Koksa, v 15,5 km vyshe pos. Sauzar 50°28'N, 84°40'E, 24 June 1991, *M. Mihailova* (ALTB; used to create Fig. 10a–e); Altai, Ust-Koksinskii r-n, d. r. Charysh, okr. p. Vladimirovka, kamenistaya step, 21 May 1982, *Doronkin* (LE).

Kazakhstan. Kazahskaya SSR, khrebet Saur, sev. makrosklon, verhovya r. Terekty bliz pos. Kyzyl-Kiya, melkozemlistye sklony s kobreziei, 16 July 1965, *V.I. Vasilevich et al*. (LE); Kazahskaya SSR, Kalbinskii khrebet, Yuzhnyi Altai, Zaisanskaya kotlovina, khrebet Saur, verhnyaya granitsa lesa i vysokogornye kobrezniki, v 3–4 km SZ Kzyl-Kiya, v 35–40 km YuV goroda Zaisan, 16 July 1965, *A.A. Yunatov* (LE).

Mongolia. Gobi-Altai aimak, Hurmin-somon, 10 km k yugo-vostoku ot Yusun-Bulaka, srednyaya chast severnogo belya, khr. Han-Taishiri, raznotravno-zhitnyakovo-kovylnaya step, 14 July 1948, *A.A. Yunatov* (LE); Hobdosskii aimak, Bulgan somon, verhovya Haragaitu gala, levoberezhnogo pritoka Buluguna, listvennichnyi les, 24 July 1947, *A.A. Yunatov* (LE); Hobdosskii aimak, Hudiirtu somon, Sairin-gol, sosna na drevnei morene, 25 July 1947, *A.A. Yunatov* (LE); Hobdosskii aimak, Bulgan somon, v 2 km k zapadu ot somona po doroge, na Haragaitu Hutul, step, 24 July 1947, *A.A. YUnatov* (LE); Gobi- Altaiskii aimak, Tamchi somon, 2–3 km yuzhnee oz. Tamchi, v shirokoi mezhgornoi doline, polynno-tipchakovaya gornaya step, 17 July 1947, *A.A. Yunatov* (LE).

China. Sintszyan-Uigurskaya avtonomnaya oblast, khrebet Saur, yuzhnyi sklon, dol. r. Karagaitu, pravoberezhnaya pad Bain-TSagan, subalpiiskii lug vyshe lesnoi granitsy, 23 June 1957, *A.A. Yunatov*, *Li Shi-in*, *Yuan I-fen* (LE).

***Festucaovina* agg.**

The aggregate comprises three species in the AM, *F.ovina*, *F.sphagnicola* and *F.kuprijanovii*.

**Notes.***Festucaovina* is easily distinguished by green or bluish green spikelets, the leaf blade cross-section with abaxial sclerenchyma in a continuous or sometimes discontinuous in 3 main islets of low profile layer, with only a midrib well defined, and with 5 or 7 vascular bundles. Within the *F.ovina* complex in the territory of the AM, two more species close to *F.ovina (F.sphagnicola* and *F.kuprijanovii)* are identified, however molecularly they form a common clade (Kriuchkova et al. 2023).

*Festucakuprijanovii* is the next species of the *F.ovina* complex. It was described by Chusovljanov (1998) based on macromorphological and anatomical data. *Festucakuprijanovii* is known only from the type locality (RUSSIA. Gornyi Altai. Ulaganskii raion, verhovya r. Nizhnyaya Koksu, lev. pritoka r. Ulusuk, sklon, na skalah, 31 July 1991, *M.V. Olonova*, *M.M. Silanteva* and Altaiskii krai, Kosh-Agachskii r-on, okrestnosti s. Kurai, pravyi bereg r. Tyute, 15 July 1982, *V.N. Kutafev*, *T. Eremina* (LE)). *Festucakuprijanovii* is close to *F.ovina* and *F.sphagnicola*. However, *F.kuprijanovii* differs from *F.ovina* in the continuous subepidermal layer consisting of 2–3 cells with a thickening opposite the midrib consisting of 5–6 cells (vs similar continuous subepidermal layer consisting of 2–3 cells), brown spikelets (vs green; Table 1). *Festucakuprijanovii* differs from *F.sphagnicola* in the continuous subepidermal layer consisting of 2–3 cells with a thickening opposite the midrib consisting of 5–6 cells (vs similar continuous subepidermal layer consisting of 2–3 cells; Chusovljanov 1998). Molecular research, however, grouped *F.kuprijanovii* and *F.ovina* into a common clade (Kriuchkova et al. 2023).

**Table 1.** Main morphological differences between species of the *F.ovina* aggregate.

Character	*F.kuprijanovii*	*F.sphagnicola*	*F.ovina*
Spikelet	brown	brown	green
Abaxial sclerenchyma	a continuous subepidermal layer thickening opposite the midrib	a continuous subepidermal layer	a continuous subepidermal layer or subcontinuous in 3 main islets of low profile
Count of chromosomes	unknown	28	14

The similar issue refers to another species of the *F.ovina* complex, *F.sphagnicola.* Some botanists treated *F.sphagnicola* as a subspecies of *F.ovina* (Tzvelev 1971, 1976; Probatova and Sokolovskaya 1980; Alexeev 1990; Lu et al. 2006), or independent species (Tzvelev and Probatova 2019). Further, Alexeev considered diploid individuals to be *F.ovina* s. str. and referred polyploid individuals to other species. Consequently, tetraploid individuals of *F.ovina* from the AM were referred to *F.sphagnicola* (Table 1). The last mentioned taxon differs from *F.ovina* by the color of spikelets (brown vs green, respectively), the elevation of occurrence (over 1700 m vs up to 1700 m, respectively), the number of chromosomes (tetraploid vs diploid).

Molecular research, however, grouped *F.sphagnicola*, *F.kuprijanovii* and *F.ovina* into a common clade (Kriuchkova et al. 2023). Thus, further studies including molecular (at the population level), morphological, and cytological analyses are needed on the above mentioned group of taxa from the entire area of their distribution to resolve whether *F.kuprijanovii* and *F.sphagnicola* are separate or conspecific with *F.ovina*.

***Festucaovina* L., Sp. Pl. 1: 73. 1753.**

= F.ovinavar.ruprechtii Boiss., Fl. Orient. 5: 619. 1884.

≡ *F.ruprechtii* (Boiss.) V. Krecz. et Bobr., Fl. SSSR 2: 507. 1934.

≡ F.ovinasubsp.ruprechtii (Boiss.) Tzvel., Bot. Zhurn. 56 (9): 1255. 1971.

= F.supinavar.elata Drobow, Tr. Bot. Muz. Akad. Nauk 153. 1915.

= *F.supina* auct. non Schur: V. Krechetovich i Bobrov, Fl. SSSR 2: 504. 1934.

**Type. *Lectotype*** (designated by Kerguélen in Lejeunia 75: 150 1975) habitat in Alpibus Lapponicae, Helvetiae, Scotiae (LINN 92.1); isolectotype (LAPP 55).

**General distribution.** The species is widely distributed, it is common in the Arctic zone of Eurasia and North America, and occurs also in mountains farther to the south of Eurasia (Kazakhstan, China, Mongolia).

**Figure 11.**
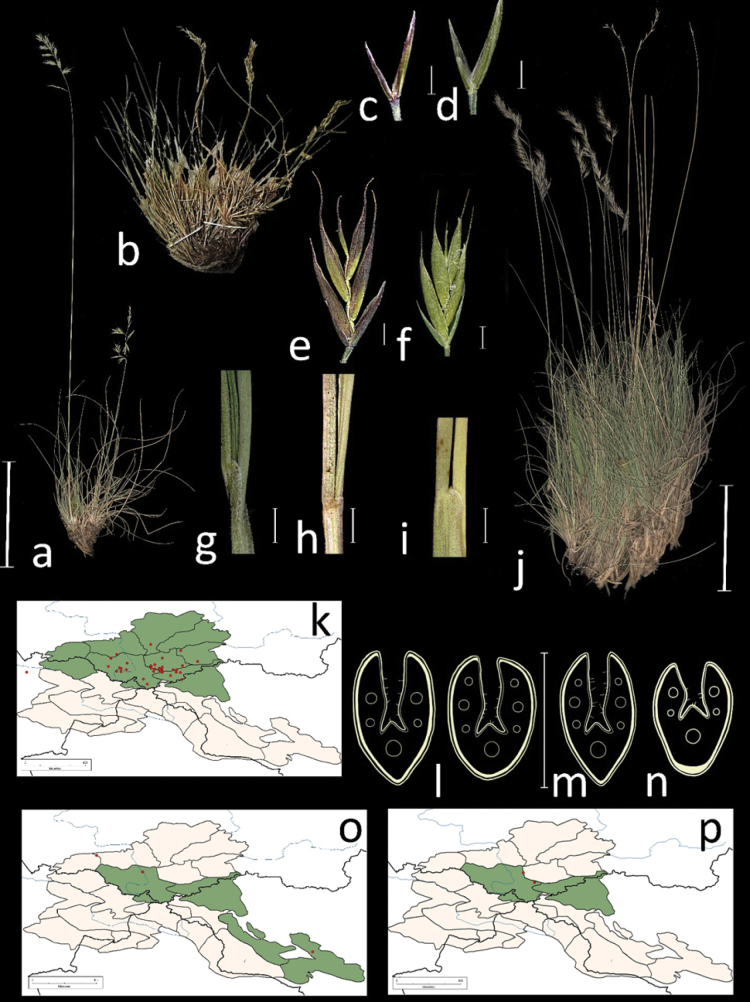
General habit of **a***F.ovina***b***F.kuprijanovii***j***F.sphagnicola* glumes, lateral view **c***F.sphagnicola***d***F.ovina* spikelets **e***F.sphagnicola***f***F.ovina* junction of leaf sheath and blade, lateral view **g***F.sphagnicola***h***F.ovina***i***F.kuprijanovii* leaf-blade cross-section **l***F.sphagnicola* (two cross-sections) **m***F.ovina***n***F.kuprijanovii* distribution maps **k***F.sphagnicola***o***F.ovina***p***F.kuprijanovii*. Scale bars: 10 cm (**a, b, j**); 1 mm (**c–i**); 0.5 mm (**l–n**). The green colour on the map refers to information on species-distribution in the region known from literature data, red points mark localities confirmed by us during revision of herbarium materials.

**Distribution in the AM.** Very rare; А3, ZM1, ZM3, UM (Fig. 11o).

**Habitat.** Forest, meadows, steppes, sand, rock fissures; elev. 500–1700 m.

**Flowering period.** May–June.

**Chromosome number.** 2*n*=14 (Altai; Probatova and Sokolovskaya 1980; Irkutskaya Oblast; Probatova et al. 2009).

**Notes.** See notes under *F.ovina* agg.

**Specimens examined.** Russia. Respublika Altai, Kosh-Agachskii raion, srednee techenie reki Ortalyk, levoberezhe, listvennichnik s mohovoi podstilkoi, elev. 2100 m, 50°07'28"N, 87°50'19"E, 5 July 2020, *E.A. Kriuchkova*, *D.D. Ryzhakova*, *P.D. Gudkova* (TK; used to create Fig. 11); Na granitse Tomsk. i Enis. Gub. Vodorazdel r.r. V.S. Lyusa i B. Usy, u lesnogo predela, 22 June 1901, *V.S. Titov* (LE).

Mongolia. Prihubs, per. Sagsain-daba k vostoku Hathyla po doroge na Chindaman-Undursomok, zabolochennyi listvennichnyi les s ernikom po grebnyu, 30 July 1972, *V. Grubov et al.* (LE); Ubsanurskii aimak, Turun somon, khr. Han-Huhoi, g. TSgan Hairhan, osokovo-kobrezievyi alpiiskii lug, 23 July 1945, *A.A. Yunatov* (LE).

***Festucakuprijanovii* Chus. Bot. Zhurn. 83: 113. 1998.**

**Type.** [Russia] Gornii Altai. Ulaganskii raion, verkhovya r. Nizhnyaya Koksu, lev. pritoka r. Ulusuk. Sklon na skalakh. 31 July 91, *Olonova*, *Silanteva* (holotype LE01011330!).

**General distribution.** Altai mountains, endemic.

**Distribution in the AM.** Very rare; А3, ZM1 (Fig. 11p).

**Habitat.** Among rocks, petrophytic steppes, elev. 1500–2500 m.

**Flowering period.** June–July.

**Chromosome number.** 2*n*=unknown.

**Notes.** See notes under *Festuca* agg *ovina*.

**Specimens examined.** Russia. Altaiskii krai. Kosh-Agachskii r-on, okrestnosti s. Kurai. Pravii bereg r. Tyute. 15 July 1982. *Kutafev V.N.*, *Eremina T.* (paratype LE!); Gornyi Altai. Ulaganskii raion, verhovya r. Nizhnyaya Koksu, lev. pritoka r. Ulusuk, sklon, na skalah, 31 July 1991, *M.V. Olonova*, *M.M. Silanteva* (LE; used to create Fig. 11); Altaiskii krai, Kosh-Agachskii r-on, okrestnosti s. Kurai, pravyi bereg r. Tyute, 15 July 1982, *V.N. Kutafev*, *T. Eremina* (LE); Respublika Altai, Kosh-Agachskii r-on, verkh. r. Kokorya, lev. bereg. 50°09'N, 88°55'E, 24 August 1995, *A.I. Shmakov*, *Dyachenko S.*, *Golyakov P.*, *Smirnov S.* (paratype has been lost SSBG).

***Festucasphagnicola* B. Keller, Zap. Voronezh. Selkokhoz. Inst. 11: 78. 1928.**

**Type. *Neotype*** (designated by Alexeev 1979: 129) [Russia] Prov. Tomsk, distr. Biisk, alpinum Karakolskii, tundra muscoso-lichenosa, 19 July 1915, *P. Krylov* (LE!).

**General distribution.** Middle (northeast) and Central (North) Asia, Russia (Eastern, Western Siberia, Sayans), Mongolia (Altai).

**Distribution in the AM.** Common; А1, А2, А3, А4, А5, А6, ZM1, KAD1 (Fig. 11k).

**Habitat.** Alpine zone, steppes, forests; elev. 1700–3500 m.

**Flowering period.** July–August.

**Chromosome number.** 2*n*=28 (Altai; Probatova and Sokolovskaya 1980).

**Notes.** See notes under *F.ovina* agg.

**Specimens examined.** Russia. Respublika Altai, Kosh-Agachskii raion, srednee techenie reki Ortolyk, levoberezhe, elev. 2300 m, alpiiskii lug na opushke listvennichnika, 50°17'59.7"N, 87°50'21.1"E, 5 July 2020, *E.A. Kriuchkova*, *D.D. Ryzhakova*, *P.D. Gudkova* (ALTB; used to create Fig. 11a, c–e); Respublika Altai, levyi bereg r. Suhoi Tytugem, ernik, elev. 2350 m, 18 July 1999, *D. Chusovljanov* (KUZ); Altai, Ust-Koksinskii raion, okr. s. Abai, verhovya r. Ayuly, alpiiskii lug, 24 June 1955, *N.T. Tzehanovskaya*, *V. Efremkov* (ALTB); Terehtinskii khrebet, dol. r. Terekty, alpiiskii lug, 1 July 1951, *G. Pavlova* (LE); Altaiskii zapovednik okr. oz. Dzhulukul. dolina r. Bogoyash, okr. gory Boksy, elev. 2170 m, 23 July 1977, *N.I. Zolotuhin*, *I. Mahatkov*, *O.N. Kozlova*, *N.D. Revushkina* (ALTB); Tomskaya gub. Biiskii u. Karakolskii belok, mohovo-lishainikovaya tundra neskolko vyshe lesnogo predela, 19 July 1901, *P.N. Krylov* (TK); Altai, Oirotiya, Ongudaiskii aimak, gornyi pereval iz loga Kulady v log Altairy, ernikovo-lishainikovaya tundra, 12 October 1936, *A.V. Kalinina*, *L.A. Sokolova et B. ShishkinK.* (LE); Gornyi Altai, Ulaganskii r-n, bassein Nizhnego Ildugema 50°18'N, 88°15'E, dolina verhnego Yasatera, myatlikovaya step, 22 July 1984, *M. Danilov*, *I. Ostanina* (NS); Gornyi Altai, Ongudaiskii r-n, elev. 1640 m, 51°10'N, 85°35'E, Seminskii peerval, polyana sredi kedrovogo lesa, 26 June 1981, *D. Shaulo* (NS); Gorno-Altaiskaya AO, Kosh-Agachskii r-n, dolina r. Uzuntotygem, 50°6'N, 80°10'E, elev. 2550 m, osochkovo-zlakovaya step na ploskoi vershine, 30 July 1982, *M. Danilov*, *N. Chernitskaya* (NS); Gornyi Altai, khr. Chihacheva, v verh. r. YUstyd, v okr. oz. Kyndykty-Hol, 49°40'N, 89°30'E, elev. 2450 m, ostepnennyi subalpiiskii lug, 30 July 1982, *I. Krasnoborov*, *L. Mironova* (NS); Gornyi Altai, Ulaganskii r-n, verhovya Oroya, 50°20'N, 88°10'E, elev. 2200 m., subalpiiskii manzhetkovyi lug po pologomu sklonu, 15 July 1984, *M. Danilov*, *N. Chalchikov* (NS); Altai, Kosh-Agachskii r-n, ushchele Kuyahtanar, elev. 1810 m, 50°10'N, 88°15'E, zabolochennaya lozhbina, 7 July 1983, *M. Danilov*, *O. Babarykina* (NS); Gornyi Altai, Kosh- Agachskii raion, verhovya Bashkausa, elev. 2400 m, 50°15'N, 89°12'E, driadovaya shchebnistaya tundra, 27 July 1983, *M. Danilov*, *L. Gunderina* (NS); Respublika Altai, Ulaganskii raion, 15 km na sever ot Aktasha, Kustarnichkovaya tundra (Kurilskii chai), 50°28'29"N, 87°37'22"E, 7 July 2020, *E.A. Kriuchkova*, *D.D. Ryzhakova*, *P.D. Gudkova* (TK); Respublika Altai, Kosh-Agachskii raion, srednee techenie reki Ortolyk, levoberezhe, elev. 2300 m, alpiiskii lug, 50°18'27"N, 87°49'18"E, 5 July 2020, *E.A. Kriuchkova*, *D.D. Ryzhakova*, *P.D. Gudkova* (TK); Respublika Altai, Kosh-Agachskii raion, kkhrebet Chikhacheva, dolina r. Karaoyuk v srednem techenii, 49°50'40"N, 89°32'30"E, 2 August 1999, *A.I. Shmakov et al.* (ALTB); Altaiskii zapovednik, okresnosti Yailyu, poberezhe Teletskogo ozera, tundra na vershine, 20 July 1945, *L. Tyulipa* (LE); YUzhnyi makrosklon Kuraiskogo hrebta, verhove r. Ortolyk, subalpiiskii lug, elev. 2000 m, 12 July 1999, *A.A. Ebel* (KUZ); Vostochnyi Altai, khr. Kurkure, r. Turanaya, elev. 2100 m, stenka kara sz eksp., v nizhnei chasti, krupnokamenistaya osyp, 10 August 1977, *L. Marina*, *Galanin A.*, *Zoloterin N.* (LE); Vost. Altai, khr. Kurkure, pravyi pritok r. B. Kurkure, sklon s-z eksp, kustarnik s ruchem, 7 August 1976, *Marina L.*, *Galanin A.*, *Zolotuhin N.* (LE); Vostochnyi Altai, khr. Kurkure, r. “Luchshaya”, elev. 2100 m, sklon yuzhn. eksp., ernik raznoravno-zlakovyi, 14 July 1976, *L. Marina*, *Galanin A.*, *Zoloterin N.* (LE).

Kazakhstan. Yuzhnyi Altai, khr. Azutau, Urunhaiskii pereval, razrezhennyi listvennichnyi les, ostepnennye lugoviny, elev. 1800 m, 20 June 1986, *Yu. Kotuhov* (ALTB); Vostochnyi Kazakhstan, Koksinskii hr., elev. 1800 m, nizkotravnye alpiiskie luga, 10 August 2004, *Yu. Kotuhov* (KUZ).

***Festucapseudovina* Hach. ex Wiesb., Osster. Bot. Zeitschr. 30: 126. 1880.**

**Type.** Im Thale der reichen Liesing zwischen Kalksburg und dem Rothen Stadel (Austria) (W).

**General distribution.** Occurs in Europe and Russia.

**Figure 12.**
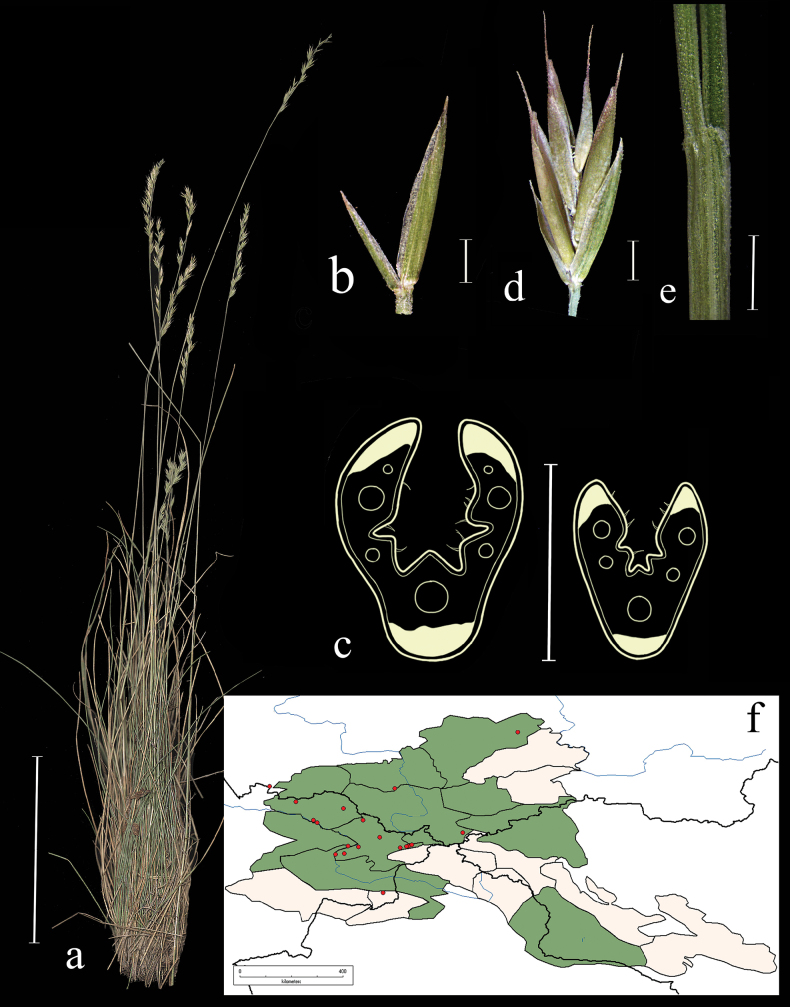
*Festucapseudovina***a** general habit **b** glumes, lateral view **c** spikelet, lateral view **d** junction of leaf sheath and blade, lateral view **e** leaf-blade cross-section **f** distribution map. Scale bars: 10 cm (**a**); 1 mm (**b–d**); 0.5 mm (**e**). The green colour on the map refers to information on species-distribution in the region known from literature data, red points mark localities confirmed by us during revision of herbarium materials.

**Distribution in the AM.** Common; А1, А2, А3, А4, ZM1, KAD1, KAD2, KAD5, KAD6, KAD9 (Fig. 12f).

**Habitat.** Steppes, meadows, forests, sand, marshes; elev. 400–3000 m.

**Flowering period.** May–July.

**Chromosome number.** 2*n*=14 (Perm region; Alexeev 1974), 2*n*=42 (Novosibirsk region; Krasnikov, 1991).

**Notes.***Festucapseudovina* is morphologically similar and closely related to *F.valesiaca*. The color of the leaves (bluish-green or green) is the only morphological character that separates these species (Alexeev 1990; Tzvelev 1976; Lu et al. 2006; Tzvelev and Probatova 2019). Most specimens previously identified as *F.pseudovina* have been re-identified by us as *F.valesiaca*, because both are bluish green but are characterised by varying colour saturation of leaf blades. In molecular research, *F.pseudovina* specimens collected from the AM were shown to be hybrids between *F.valesiaca* and *F.rupicola* (Kriuchkova et al. 2023). The species needs further study, including molecular (at the population level), morphological, and cytological analyses.

**Specimens examined.** Russia. Altaiskii krai, Bystroistokskii raion, 8 km na yug ot s. Priobskoe, raznotravno-zlakovyi lug na sklone (*Fragariaviridis*, *Irisruthenica*, *Stipa* sp., *Poa* sp., *Achillea* sp., *Artemisiagmelinii*), 52°18'01"N, 84°25'58"E, 13 June 2020, *P.D. Gudkova*, *Zolotov D.V.*, *E.A. Kriuchkova* (ALTB; used to create Fig. 12a–e); Respublika Altai, Ust-Kanskii raion, okolo sela Vladimirovka. Yuzhnyi kustarnikovyi kamenistyi sklon, pravyi bereg reki Charysh (*Caraganafrutex*, *Spirea* sp., *Achnatherum* sp., *Stipa* sp., *Orostachysspinosa*), 51°3'15"N, 84°11'26"E, 4 June 2020, *E.A. Kriuchkova*, *D.D. Ryzhakova*, *P.D. Gudkova* (ALTB).

Kazakhstan. Yuzhnyi Altai, khrebet Sarym-Sakty, yuzhnyi bort Karakabinskoi vpadiny, yugo-vostochnyi sklon, 11 July 1991, *Yu. Kotuhov* (KUZ); Khr. Kalbinskii, v raione s. Novo-Timofeevka, vyrovnennye peski, kovylnaya step, 18 June 1983. *Yu. Kotuhov* (KUZ); Yuzhnyi Altai, khr. Narymskii, v raione s. Sergeevka, yugo-zapadnyi shchebnistyi sklon, elev. 800 m, 31 July 1986, *Yu. Kotuhov* (KUZ); Yuzhnyi Altai, khr. Kurchumkii, vostochnye otrogi, elev. 1700 m, dolina r. Tautekeli, ostepnennye luga, 3 August 1985, *Yu. Kotuhov* (KUZ); Vostochno-Kazakhstanskaya obl., Kurchumskii r-n., 10 km severnee p. Kuigan v raione Kaznakovskoi perepravy, yugo-zapadnye predgorya khr. Narymskii, na kamnyah, 8 June 1998, *D.V. Chusovljanov* (KUZ); VKO, okr. s. Medvedki, khr. Narymskii, suhodolnyi lug, vershina gory, 15 July 1978, *Kupriyanov A.N.* (KUZ); Zapadnyi Altai, khr. Narymskii, uroch. Terekty, v raione s. Novo-Berezovka, elev. 1600 m, ostepnennye alpiiskie luga, 30 July 1970, *Yu. Kotuhov* (KUZ); Zapadnyi Altai, khr. Ivanovskii, v raione s Poperechnoe, dolina r. Belaya Uba, ostepnennyi lug, 25 July 1970, *Yu. Kotuhov* (KUZ); Yuzhnyi Altai, khr. Narymskii, sev-zap. Otrogi, v raione s. Sergeevki, vershina grivy, shchebnistye uchastki, 11 June 1988, *Yu. Kotuhov* (KUZ); Vostochno-Kazakhstanskaya obl., Kurchumskii r-n, gory Bukumbai, kamenistoe ushchele, zlakovaya step, 9 June 1998, *D.V. Chusovljanov* (KUZ); Yuzhnyi Altai, khr. Tarbagatai, Karakabinskaya vpadina, elev. 1600 m, ostepnennye luga, 2 August 1985, *Yu. Kotuhov* (KUZ); Zaisanskaya kotlovina, Bukonskie peski, uste r. Bukoni, vyrovnennye pesky, 8 June 1970, *Yu. Kotuhov*; Altai, Ust-Kamenogork, na sklone v dol. r. Ulby, 2 June 1931, *B. ShishkinK.*, *L. Chilikina i Sumnevich G.* (LE); Kazahskaya ASSR, Ust-Kamenogorsk, gora Tarabachiha, tipchakovo-kelerievyi sklon k r. Irtyshu, 1 June 1931, *Shishkin K.B.*, *Sumnevich G.* (LE); Khr. Manrak, v raione s. Priozernoe, kustarnikovo-zlakovaya step, 4 June 1976, *Yu. Kotuhov* (KUZ); Khr. Saur., dolina r. Terekty, ugolnyi karer, uglistye slantsy, 23 June 1986, *Yu. Kotuhov* (KUZ).

***Festucapseudosulcata* Drobow, Tr. Bot. Muz. Akad. Nauk 14: 156. 1915.**

**Type. *Lectotype*** (designated by Tzvelev 1968: 168). [Russia] Vilyuiskii okr., r. Chona v 35 km vyshe Ustya ee pritoka Yagody, 30 July 1914, N°556, *V. Drobov* (LE!).

**General distribution.** Russia (Eastern Siberia, Far East), North-Eastern China, Mongolia.

**Figure 13.**
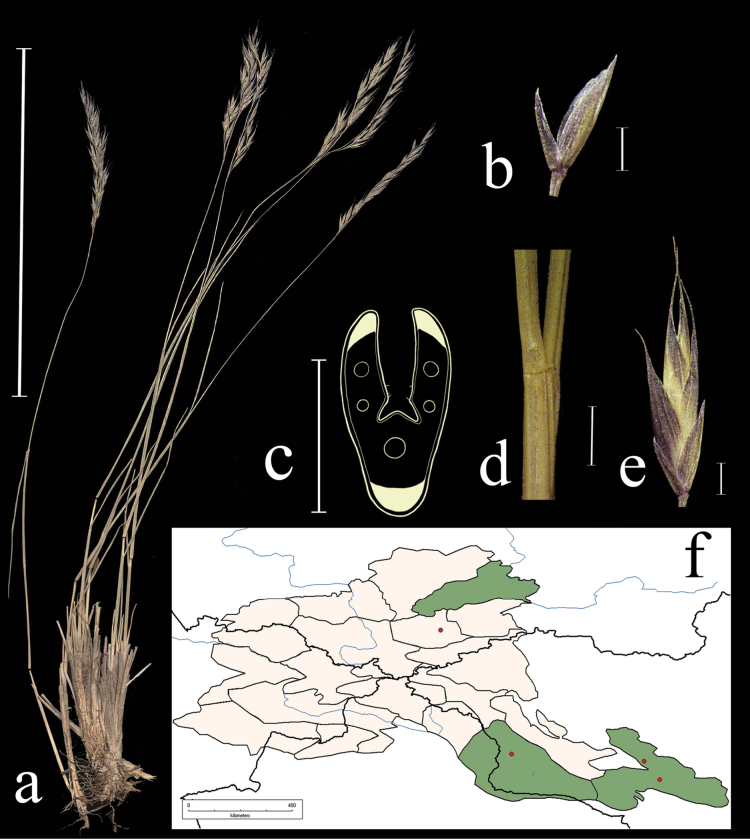
*Festucapseudosulcata***a** general habit **b** glumes, lateral view **c** leaf-blade cross-section **d** junction of leaf sheath and blade, lateral view **e** spikelet, lateral view **f** distribution map. Scale bars: 10 cm (**a**); 1 mm (**b, d, e**); 0.5 mm (**c**). The green colour on the map refers to information on species-distribution in the region known from literature data, red points mark localities confirmed by us during revision of herbarium materials.

**Distribution in the AM.** Very rare; А5, KAD9, UM (Fig. 13f).

**Habitat.** Steppes, stony slopes, rocks, forests; elev. 1000–2500 m.

**Flowering period.** June–July.

**Chromosome number.** 2*n*=28 (Krasnoyarsk territory, Amur region; Alexeev et al. 1988).

**Notes.** Morphologically, *F.pseudosulcata* belongs to the *F.valesiaca* group. *Festucapseudosulcata* differs from *F.rupicola* in the number of ribs in a leaf blade cross-section (one well-defined midrib vs 3 well-defined ribs), the leaf sheaths of tillers (fused for ¼–¹⁄3 their length vs fused for ¹⁄6 their length).

**Specimens examined.** Russia. Respublika Altai, Kosh-Agachskii raion, Kuraiskii hr., verhnee techenie r. Kokorya, prav. bereg, skl.vost. ekspozitsii, scaly, 50°06,5'N, 88°52'E, 27 June 1993, *R.V. Kamelin*, *A.I. Shmakov*, *P.V. Golyakov*, *A.Ya. Kiselev*, *T.V. Krestovskaya*, *M. Kashcheev*, *M.A. Mihailova*, *A. Solovev* (KUZ; used to create Fig. 13a–e); Khakasiya, Shirinskii r-n, okr. p. Sarala, stepnoi sklon, 1984, Rybinskaya (LE).

Mongolia. Gobi-Altai aimak, Hurmin-somon, 10 km k yugo-vostoku ot Yusun-Bulaka, srednyaya chast severnogo belya, khr. Han-Taishiri, raznotravno-zhitnyakovo-kovylnaya step, 14 July 1948, *A.A. Yunatov* (LE); Gobi-Altai aimak, Tumun somon, severnyi sklon khr. Han-Taishiri, pyatno listvennichnogo lesa km v 15 k yu-v ot Yusun-Bulaha, osokovo-tipchakovyi lug, 11 September 1948, *V.I. Grubov* (LE).

***Festucarupicola* Heuff., Verh. Zool.-Bot. Ges. Wien 8: 2335. 1858.**

= *F.ganeschinii* Drobow, Tr. Bot. Muz. Akad. Nauk 14: 175. 1915.

=*F.recognita* Reverd., Sist. Zametki Mater. Gerb. Krylova Tomsk. Gosud. Univ. Kuybysheva 3–4: 7. 1928.

=F.ovinasubsp.sulcatavar.genuina Hack., Monogr. Festuc. Europ. 104. 1882.

=F.valesiacasubsp.sulcatavar.hirsuta (Link) E. Alexeev, Byulleten MOIP. Otdel Biologicheskii 78 (3): 109. 1973.

**Type.** In rupestribus umbrosis montis Domugled ad Thermas Herculis (holotype W).

**General distribution.** Wide distribution species from Europe, to south Central Siberia.

**Figure 14.**
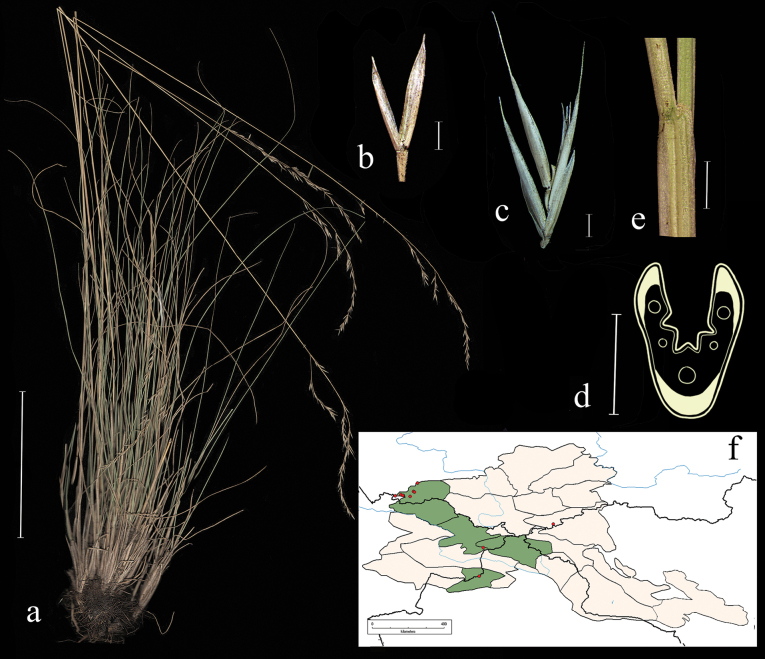
*Festucarupicola***a** general habit **b** glumes, lateral view **c** spikelet, lateral view **d** junction of leaf sheath and blade, lateral view **e** leaf-blade cross-section **f** distribution map. Scale bars: 10 cm (**a**); 1 mm (**b, c, e**); 0.5 mm (**e**). The green colour on the map refers to information on species-distribution in the region known from literature data, red points mark localities confirmed by us during revision of herbarium materials.

**Distribution in the AM.** Very rare; А2, KAD1, KAD4, KAD6, KAD7, KAD9 (Fig. 14f).

**Habitat.** steppes, forests, rock, limestones, solonetzs; elev. 300–2500 m.

**Flowering period.** May–June.

**Chromosome number.** 2*n*=42 (Ukraine; Alexeev et al. 1988).

**Notes.***Festucarupicola* is rare in the AM, and Western Siberia. It occurs here at its easternmost distribution limit. *Festucarupicola* is characterised by green leaf blades; the leaf blade cross-section with three sclerenchyma strands, with 5 or 7 vascular bundles; the leaf blades (0.45)0.55–0.85(1.1) mm wide; the leaf sheaths of tillers fused for ¹⁄6 their length; the spikelets green; the lemma (4.5)4.8–5(6) mm in length; the spikelets (5.5)6.5–8.5(10) mm in length.

Recent molecular research revealed that *F.rupicola* belongs to the *F.valesiaca* group but forms a separate subclade (Kriuchkova et al. 2023).

**Specimens examined.** Russia. Altaiskii krai, Zmeinogorskii raion, okr. oz. Kolyvanskoe, na granitnyh ostantsah, 14 June 1999, *O. Maslova*, *D. Chusovljanov* (ALTB, KUZ; used to create Fig. 14a–e);Altaiskii krai, Loktevskii r-n, 2 km severo-vostochnee s. Pokrovka, sopochnyi massiv, kamenistyi ostepnennyi sklon, 20 June 2001, *O.M. Maslova*, *A.V. Grebenyuk*, *D.V. Chusovljanov* (ALTB); Altaiskii krai, Loktevskii r-n, 3 km severo-vostochnee s. Ustyanka, sopochnyi massiv, shchebnisto-glinistyi sklon yuzhnoi ekspozitsii, 20 June 2001, *O.M. Maslova*, *A.V. Grebnyuk*, *D.V. Chusovljanov* (ALTB); Altaiskii krai, Loktevskii raion, okr. s. Ustyanka, melkosopochnyi massiv SV sela, 51°10'N, 81°38'E, 14 August 1998, *T.O. Strelnikova*, *O.M. Maslova*, *D.V. Chusovljanov*, *L. Sokolova* (ALTB); Altaiskii krai, Zmeinogorskii raion, okr. oz. Kolyvanskoe, Yuzhnyi bereg, sklon severnoi ekspozitsii 51°21'N, 82°12'E, 25 June 1996, *A.N. Kupriyanov*, *E.V. Samusenko* (KUZ); Altaiskii krai, Loktevskii raion, 2 km severnee pos. Removskii, sopochnyi massiv, stepnoi sklon, 20 June 2001, *O.M. Maslova*, *A.V. Grebenyuk*, *D.V. Chusovljanov* (ALTB); Altaiskii krai, Tretyakovskii r-n, okr. s. Kraboliha, kamenistyi ostepnennyi sklon sopki, na kamnyah, 22 June 2001, *O.M. Maslova*, *A.V. Grebenyuk*, *D.V. Chusovljanov* (ALTB); Tuvinskaya ASSR, Ulug-Hemskii r-n, Zap. Sayan, Uyukskii hr., sr. tech. r. Arty-Haya, prav. prit. r. Vayan-Kol, yuzhn. sklon. ovsyantsovo-kovylno-osochkovaya step, 5 July 1975, *M. Lomonosova*, *T. Grushevskaya* (NS); Altai, Kosh-Agachskii aimak, Chuiskaya kotlovina, okr. pos. Tashanty, step, 20 July 1931, *A. Kuminova*, *A. Skvortsova* (LE); Altaiskii krai, Zmeinogorskii r-n, okr. s. Savvushka, v rasshcheline skaly, 8 June 1983, *Frizen*, *Zuev* (NSK); Altaiskii krai, Zmeinogorskii raion, okr. oz. Kolyvanskoe, na granitnyh ostantsah, 14 June 1999, *O.M. Maslova*, *D.V. Chusovljanov* (KUZ); Altaiskii krai, Kurinskii raion, 2 m vverh po lev. ber. r. Charysh ot byvsh. s. Vostruha, 51°48'N, 82°22'E, 16 June 1999, *R.V. Kamelin et al.* (ALTB); Altaiskii krai, Smolenskii raion, v 5 km ot sela Berezovo na severo-vostok po doroge, lugovaya kovylnaya step na yugo-zapadnom sklone (*Stipa* sp., *Potentilla* sp., *Delphinium* sp., *Irisruthenica*, *Festuca* sp., *Poa* sp., *Bromusinermis*), 51°48'27"N, 82°22'19"E, 6 June 2020, *E.A. Kriuchkova*, *D.D. Ryzhakova*, *P.D. Gudkova* (TK).

Kazakhstan. Yuzhnyi Altai, khr. Azutau, Uspenskaya vpadina, dolina r. Yuelezek, ostepnennye luga, 27 July 1983, *Yu. Kotuhov* (KUZ); Khr. Saur, vodorazdel rek B. i M Dzhemeneya, kovylno-raznotravnaya step, 6 July 1930, *N. Goncharov*, *A. Borisova* (KUZ).

***Festucasaurica* E. Alexeev, Novosti Sist. Vyssh. Rast. 21. 1976.**

**Type.** Kazakhstan, khr. Saur, sev. sklon, verkhovya r. Terekty bliz pos. Kyzyl-Kiya, travyanistye sklony s sibbaldiei, 16 July1965, V. Vasilevich, Z. Karamysheva, N. Nikolskaya, E. Rachkovskaya, I. Safronova (holotype and isotype LE!).

**General distribution.** Saur ridge, endemic of Altai.

**Figure 15.**
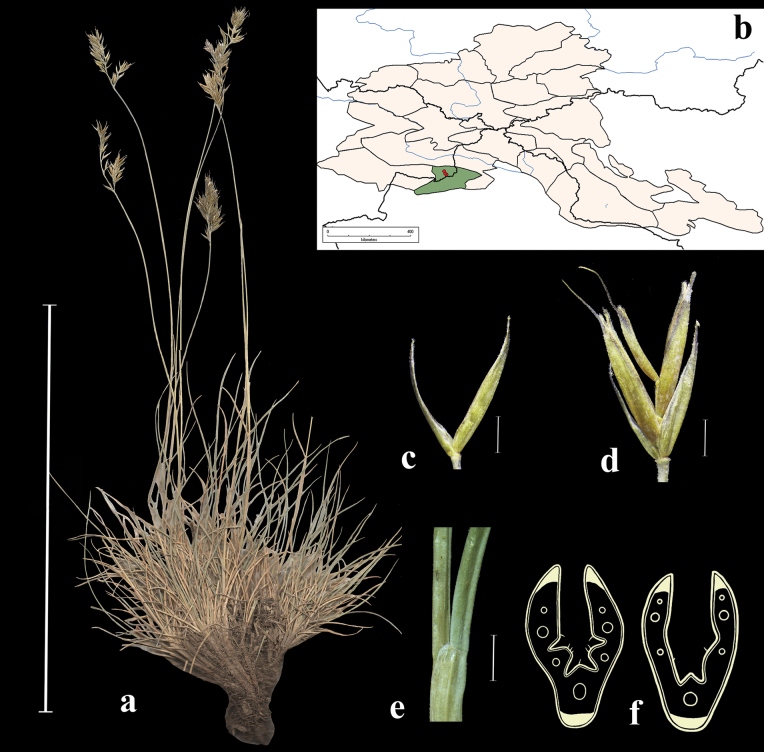
*Festucasaurica***a** general habit **b** distribution map of the AM**c** glumes, lateral view **d** spikelet, lateral view **e** junction of leaf sheath and blade, lateral view **f** leaf-blade cross-section. Scale bars: 10 cm (**a**); 1 mm (**c–e**); 0.5 mm (**f**). The green colour on the map refers to information on species-distribution in the region known from literature data, red points mark localities confirmed by us during revision of herbarium materials.

**Distribution in the AM.** Very rare; KAD4 (Fig. 15d).

**Habitat.** Forests, alpine zone; elev. 2000–2600 m.

**Flowering period.** June–July.

**Chromosome number.** 2*n*=unknown.

**Notes.***Festucasaurica* is an endemic species of the AM. The species is highly variable in morphology: the number of vascular bundles varies from 5 to 7; the leaf blade cross-section may have only one single midrib or two additional lateral well-defined ribs; the shape of ribs varies from rounded to triangular; the shape of the leaf blade cross-section varies from obovate with an elongated keel to wide-lanceolate; the leaf sheaths of tillers may be fused for ½–¾ their length; the abaxial surface of the leaf blade is characterised as glabrous or scabrous; and shoots are either grouped by 2–3 and surrounded by a cover of old sheaths. According to our molecular research, *Festucasaurica* is separated in an independent clade (Kriuchkova et al. 2023).

**Specimens examined.** Kazakhstan. Khr. Saur, verh. r. Kyzyl-Kiya, sev.-zap. sklon, razrezhennyi listvennichnyi les, ostepnennyi alpiiskie luzhaiki, elev. 2000 m, 16 July 1992, *Yu. Kotuhov* (KUZ; used to create Fig. 15a, c–f); Khr. Saur, verh. r. Kyzyl-Kiya, kamenistaya tundra, elev. 2600 m, 14 August 1991, *Yu. Kotuhov* (KUZ, 3 sheets); Kkhrebet Saur, verhovya r. Kyzyl-Kiya, severo-zapadnyi sklon, elev. 2000 m, razrezhennyi listvennichnyi les, ostepnennyi alpiiskii lug, 16 July 1992, *Yu. Kotuhov* (KUZ); Kazahskaya SSR, khrebet Saur, sev. makrosklon, verhovya r. Terekty bliz pos. Kyzyl-Kiya na meklozemistom sklone, soobshchestvo tipchaka, 16 July 1965, *V.I. Vasilevich*, *Z.V. Karamysheva*, *N.I. Nikolskaya*, *E.I. Rachkovskaya*, *I.N. Saphronova* (LE); Kazahskaya SSR, khrebet Saur, sev. makrosklon, verhovya r. Terekty, bliz pos. Kyzyl-Kiya, dresvyanistye sklony s sibbaldiei, 16 July 1965, *V.I. Vasilevich*, *Z.V. Karamysheva*, *N.I. Nikolskaya*, *E.I. Rachkovskaya*, *I.N. Saphronova* (LE).

***Festucatschujensis* Reverd., Sist. Zametki Mater. Gerb. Krylova Tomsk. Gosud. Univ. Kuybysheva 3: 3. 1936.**

**Type.** [Russia] Chuiskaya step, na vysokom beregu reki Tarkhatty, vyshe sliyaniya ee s Chegan-Burgazy, v kovylno-polynnoi stepi, 6 July 1935, *I. Plotnikov* (holotype ТK!).

**General distribution.** Kazakhstan (north of east Kazakhstan region), north Mongolia, Russia (south Siberia). South Siberia is the disjunct northernmost limit in the geographical distribution of *F.tschujensis*.

**Figure 16.**
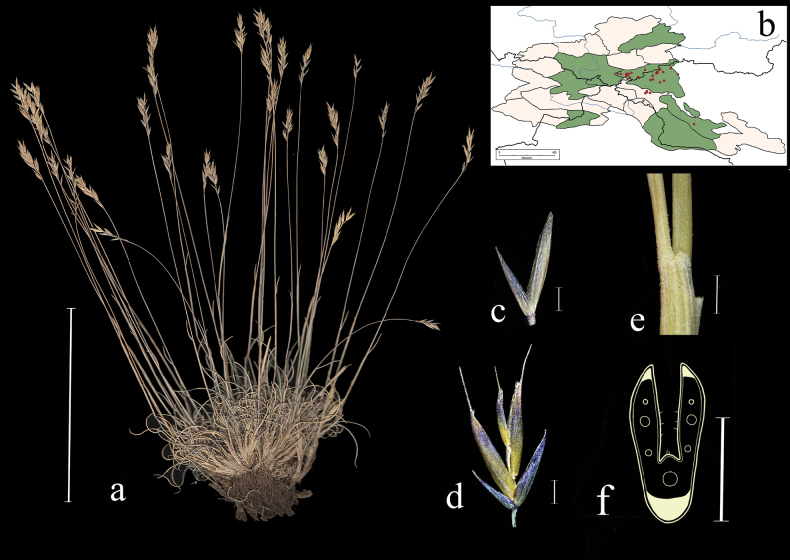
*Festucatschujensis***a** general habit **b** distribution map of the AM**c** glumes, lateral view **d** spikelet, lateral view **e** junction of leaf sheath and blade, lateral view **f** leaf-blade cross-section. Scale bars: 10 cm (**a**); 1 mm (**c–e**); 0.5 mm (**f**). The green colour on the map refers to information on species-distribution in the region known from literature data, red points mark localities confirmed by us during revision of herbarium materials.

**Distribution in the AM.** Common; А3, А5, ZM1, ZM2, ZM3, KAD4, KAD8, KAD9 (Fig. 16b).

**Habitat.** Petrophytic steppes, stony slopes, rock fissures; elev. 2000–3500 m.

**Flowering period.** June–July.

**Chromosome number.** 2*n*=28 (Altai Republic; Agapova et al. 1993).

**Notes.** The species is easily distinguished by its lemmas 4.5–6 mm long, awns 1.5–4 mm long, leaf blades 0.5–1.1 mm wide, leaf blade cross-section with 3 well-defined ribs, leaf sheaths of tillers fused for ¹⁄3–½ their length; shoots are either grouped by 2–3 and surrounded by a cover of old sheaths. Our recent molecular research revealed that this species might hybridise with the *F.kryloviana* group (Kriuchkova et al. 2023). However, there are no morphological characters distinguishing the hybrids from *F.tschujensis*.

Alexeev (1981) and Chusovljanov (2007) mention that *F.lenensis* grows in the Mongolian part of the AM, and the species differs from *F.tschujensis* by the leaf blade structure (width of middle sclerenchyma strand is similar to lateral strands vs middle sclerenchyma strand is two-three times wider than lateral strands; leaf blades arcuate vs flexuose, respectively). However, during our revision, all the available specimens of *F.lenensis* from the AM were identified by the first author of this paper as *F.tschujensis*. Thus, the presence of *F.lenensis* in the AM needs to be confirmed.

**Specimens examined.** Russia. Altai, Kosh-Agachskii raion, dol. r. Tarhata okolo perevalochnoi bazy kolhoza im. Lenina, elev. 2150 m, 49°45'N, 88°30'E, 9 June 1982, *M. Lomonosova*, *N. Timukina* (LE; used to create Fig. 16a, c–f); Altai, Oirotiya, Koshagachskii aimak, Chuiskaya step, Kyzylchin, kamenistyi sklon, 1 August 1937 (LE); Altai, Oirotiya, Koshagachskii aimak, dol. r. Tarhatty, poimennaya terrasa, galechnikovye otlozheniya, 24 August 1936, *A.V. Kalinina*, *L.A. Sokolova*, *B.K. Shishkin* (LE); Altai, Oirotiya, Chuiskaya step, nizove r. Kok-Uzek, 16 September 1937, *Akashimin* (LE); Altai, Kosh-Agachskii r-n, dol. r. Tarhata vblizi perevalochnoi bazy kolhoza im. Lenina, raznotravno-zlavkovaya kamenistaya step sredi valunov, elev. 2300 m, vost. sklon, 49°45'N, 88°30'E, 9 June 1982, *M. Lomonosova*, *N. Timukina* (NS); Altai, Kosh-Agachskii raion, pravyi bereg r. Kyzylchin, kamenistaya step po grebnyu shleifa, 13 August 1981, *M. Lomonosova*, *N. Kilinina* (NS); Respublika Altai, Kosh-Agachskii r-n, okr. oz. Kara-Kol, kamenistye osypi, elev. 2659 m, 49°54'05"N, 87°56'51"E, 7 July 2006, *I. Han*, *R. Shtengauer* (NSK); Altai, Kosh-Agachskii r-n, r. Tarhata, raznotravno-zlakovaya step, 8 July 1982, *N. Frizen*, *V. Doronkin* (NSK); Altai, Kosh-Agachskii r-n, r. Tarhata, vblizi perevalochnoi bazy kolhoza im. Lenina, yu-z sklon, zadernovannaya kamenistaya osyp, elev. 2200 m, 49°45'N, 88°30'E, 8 July 1982, *M. Lomonosova*, *A. Vanyaev* (NS); Respublika Tuva, Mugur-Aksynskii r-on, dol. r. Mogen-Buren u ustya ruch. Bashky-Kara-Syg, 50°10'N, 89°46'E, 4 July 1995, *R.V. Kamelin*, *et al.* (ALTB); Respublika Altai, Kosh-Agachskii r-n, ushchele reki Chagan-Burgazy v srednem techenii, 49°39'N, 88°44'E, 1 August 1998, *R.V. Kamelin et al.* (ALTB); Talduair, zlakovo-kovylnaya step, elev. 2300 m, 8 August 1997, *Ebel* (ALTB); Dzhirialant, Gurvan-Ulleusal, na skalah s-z ekspozicii, subalpiiskii lug (ALTB); Respublika Altai, Kosh-Agachskii r-n, Chuiskaya step, dol. r. Bar-Burgazy bliz vyhoda iz gor, elev. 1990–2100 m, 49°50'N, 89°11'E, 20 July 2000, *R.V. Kamelin et al.* (ALTB).

Mongolia. Tolbonur-Ulgii, zlakovo-polynno raznotravnaya step, elev. 2300 m, 8 August 1997, *A.A. Ebel* (KUZ); Ubsunurskii aimak, v 48 km k Yugu ot somon Taryalan, ravnina, sev.-zap. chast uroch. Kalhat-Chessen, elev. 1950 m, kserofitnoraznotravno-zhitnyakovo-tyrsovaya step, 19 July 1980, *Z.V. Karamysheva*, *I.Yu. Sumerina*, *U. Beket*, *H. Buyan-Orshih* (LE); Ubsunurskii aimak, poima r. Tes v 60 km VSV ot Tes-somona, soobshchestvo s gospodstvom Leymus po suhim grivam, 17 June 1978, *Z.V. Karamysheva*, *I.Yu. Sumerina*, *U. Beket*, *H. Buyan-Orshih* (LE); Dzabhanskii aimak v 25 km Z ot somona Erdene-Hairhan, zlakovo-tyrsovaya step s uchastiem polukustarnichkov, 30 June 1980, *Z.V. Karamysheva*, *I.Yu. Sumerina*, *U. Beket*, *H. Buyan-Orshih* (LE); Ubsunurskii aimak, v 30 km k SSV ot Umne-gobi, polynno-petrofitnoraznotravnoe tipchakovaya step na sklone sev. eksp. elev. 2000 m, 21 June 1978, *Z.V. Karamysheva*, *I.Yu. Sumerina*, *U. Beket*, *H. Buyan-Orshih* (LE); Bayan-Ulgiiskii aimak, v 12 km k YuYuV ot somona Tolbo, melkosopochnik iz slantsev, polynno-zlakovaya step, 30 July 1980, *Z.V. Karamysheva*, *I.Yu. Sumerina*, *U. Beket*, *H. Buyan-Orshih* (LE); Ubsunurskii aimak, sopki v 33 km k Yugu ot shaht Urhai-Suren, polynno-melkodernovinnozlakovaya step v doline, 1 July 1977, *Z.V. Karamysheva*, *I.Yu. Sumerina*, *U. Beket*, *H. Buyan-Orshih* (LE); Ubsunurskii aimak, v 42 km k SV ot Buh-Murena, melkosopochnik po sev. okraine Achit-Kursk. vpadiny, tipchakovaya step, elev. 2100 m, 8 July 1978, *Z.V. Karamysheva*, *I.Yu. Sumerina*, *U. Beket*, *H. Buyan-Orshih* (LE); Kobdoskii aimak, khr. Mongolskii Altai, pereval Baga-Ulan-Daba, alpiiskii lug, elev. 2900 m, 46°41'N, 92°17'E, 6 July 2009, *I.A. Sherin*, *P.A*. *Shalimov* (LE); Zapadnaya Mongoliya, Kobdoskii aimak, Mongolskii Altai, Vostochnyi makrosklon khr. Munkh-Khayrkhan, basseyn r. Dolon-Nuryn-gol bliz severnoy chasti lednika Munkh-Khayrkhan, elev. 3300 m, 17 August 1991, *G.N. Ogureyeva*, Det. E.A. Kriuchkova (W0170756)

***Festucavalesiaca* Schleich. ex Gaudin., Agrost. Helv. 1: 242. 1811.**

**Type.**Festuca glauca marit ? Vill. De Branson (holotype LAU); in Valesia prope Branson, in apricis, Gaudin (syntype LE!).”

**General distribution.** Common in Eurasia, rare in North America (adventive).

**Figure 17.**
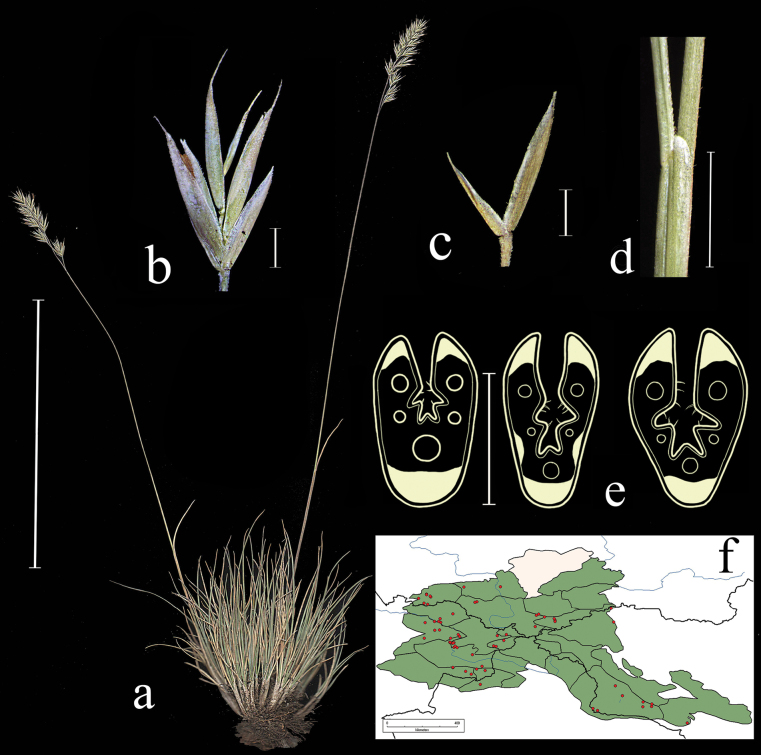
*Festucavalesiaca***a** general habit **b** spikelet, lateral view **c** glumes, lateral view **d** leaf-blade cross-section **e** junction of leaf sheath and blade, lateral view **f** distribution map. Scale bars: 10 cm (**a**); 1 mm (**b–d**); 0.5 mm (**e**). The green colour on the map refers to information on species-distribution in the region known from literature data, red points mark localities confirmed by us during revision of herbarium materials.

**Distribution in the AM.** Widespread; KAD1, KAD2, KAD3, KAD4, KAD5, KAD6, KAD7, KAD8, KAD9, А1, А2, А3, ZM1, ZM2, ZM3, UM (Fig. 17f).

**Habitat.** Steppes, among bushes, rarely deserts, meadows, stony slopes, limestones, sands, fellfield, pine forest; elev. 300–3000 m.

**Flowering period.** May–June.

**Chromosome number.** 2*n*=14 (Russia, Altai; Probatova and Sokolovskaya 1980; Primorskyi kray, Vladivostok; Probatova et al. 1991).

**Notes.***Festucavalesiaca* is a polymorphic widespread species. There is considerable variation in plant height (10–40 cm), width of tiller leaf blades (0.4–0.7 mm), length of the lemma (2.8–4.6 mm), awn length (0.5–2.2 mm), and number of florets in spikelets (3–6). The most significant diagnostic characters for *F.valesiaca* are bluish wax covering the entire plant, green spikelets, and three similar sclerenchyma strands in the leaf blade cross-section (Tzvelev 1976; Alexeev 1990; Lu et al. 2006; Tzvelev and Probatova 2019). According to our molecular studies, *F.valesiaca* includes two genetic groups: one from mountains and another from lowland (Kriuchkova et al. 2023). The species needs further study, including molecular (at the population level), morphological, and cytological analyses.

**Specimens examined.** Russia. Respublika Altai, Kuraiskii khr. r. Ortolyk, zakustarennyi sklon, 2 July 1999, A.A. Ebel (KUZ; used to create Fig. 17a–e); Altaiskii krai, Zmeinogorskii r-n, 6 km na severo-vostok ot p. Cherepanovskii, podnozhie gory Shish, step, 19 June 1999, *Usik N.A.*, *Strelnikova T.O.*, *Mungalov E.A.* (ALTB); Altaiskii krai, Kurinskii r-n, 5 km k yu-vost. ot d. Kalmytskie Mysy, dolina r. Charysh, 16 June 1999, *Usik N.A.*, *Strelnikova T.O.*, *Mungalov E.A.* (ALTB); Altaiskii krai, Soloneshenskii r-n, okrestnosti s. Sibiryachiha, 3 km k vostoku. 51°44'N, 84°07'E, 17 June 1997, *Strelnikova T.O.*, *Maslova O.M.*, *German D.A.* (ALTB); Altaiskii krai, Zmeinogorskii r-n, okr. s. Savvushka, rashchelina skaly, 8 June 1983, *Doronkin*, *Rybinskaya* (ALTB); Altaiskii krai, okresnosti poselka Konyuhi, zlakovyi stravlennyi lug (*Festuca*, *Poa*, *Stipa*, *Leymus*, *Koeleria*, *Astragalus*, *Agropyron*), 53°10'10"N, 83°42'28"E, 20.05.2020, *E.A. Kriuchkova*, *D.D. Ryzhakova*, *P.D. Gudkova* (TK); Respublika Altai, Ulaganskii raion, pereval Katu-Yaryk, vysokotravnyi subalpiiskii lug, 50°3'53"N, 87°52'11"E, 7 July 2020, *E.A. Kriuchkova*, *D.D. Ryzhakova*, *P.D. Gudkova* (TK); Respublika Altai, Kosh-Agachskii raion, srednee techenie Ortolyk, levoberezhe, elev. 2100 m, petrofitnaya step, 50°17'33"N, 87°50'53"E, 5 July 2020, *E.A. Kriuchkova*, *D.D. Ryzhakova*, *P.D. Gudkova* (TK); Respublika Altai, Kosh-Agachskii raion, 4 km zapadnee sela Kurai, petrofitnaya kovylnaya step (*Stipacapillata*, *S.orientalis*, *S.glareosa*, *S.krylovii*), 50°14'13"N, 87°50'45"E, 4 July 2020, *E.A. Kriuchkova*, *D.D. Ryzhakova*, *P.D. Gudkova* (TK); Respublika Altai, Ust-Kanskii raion, selo Ust-Kan, kamenistyi tipchakovyi sklon (*Poa* sp., *Koeleria* sp., *Orostachysspinosa*, *Festucavalesiaca*), 50°56'33"N, 84°46'40"E, 5 June 2020, *E.A. Kriuchkova*, *D.D. Ryzhakova*, *P.D. Gudkova* (TK).

Kazakhstan. Vostochno-Kazakhstanskaya obl., Zaisanskii r-n, khr. Saikan, Uroch. Koksoldy, zlakovo-raznotravnyi lug, elev. 1650 m, 14 June 1998, *D.V. Chusovljanov* (KUZ); Vostochno-Kazakhstanskaya obl., Zaisanskii r-n, khr. Saikan, Uroch. Koksoldy, peschano-kamenistyi sklon zap. ekspoz. elev. 2100 m, 14 June 1998, *D.V. Chusovljanov* (KUZ); Vostochno-Kazakhstanskaya obl., Samarskii r-n, 10 km yu-v s. Kaznakovka, Kulundzhunskie peski, bugristye peski, 7 June 1998, *D.V. Chusovljanov* (KUZ); Khr. Saur, pereval Saikan, elev. 1800 m, severnyi sklon, zlakovye ostepnennye luga, 17 June 1976, *Yu. Kotuhov* (KUZ) Khr. Manrak, gory Katan-Chilik, elev. 800 m, vershina grivy, kamenistaya zlakovaya step, 15 July 1985, *Yu. Kotuhov* (KUZ); Yuzhnyi Altai, khr. Kurchumskii, dolina r. Tautkeli, ostepnennye luga, elev. 1700 m, 3 August 1985, *Yu. Kotuhov* (KUZ); Kazahskaya ASSR, Ust-Kamenogorsk, 1931, *B. Shishkin*, *G. Sumnevich* (LE); Yuzhnyi Altai, khr. Yuzhnyi Altai, verh. r. Kara-Koba, u grebnya, alpiiskii poyas, elev. 2500–2800 m, 3 August 1987, *A. Ivashchenko* (KUZ); Khr. Kalbinskii, v raione s. Podgornoe, predgornye stepi, 19 June 1983, *Yu. Kotuhov* (KUZ).

Mongolia. Hobdosskii aimak, Bulgan somon, Mongolskii Altai, levoberezhnyi sklon v dolinu Inderzhin gola, u letnei stoyanki somona, kamenistaya step, 25 August 1947, *A.A. Yunatov* (LE); Gobi-Altai aimak, Altai somon, mezhdu khr. Adzhi-Bogdo i Alak-Hairhan u perevala v verhovyah Tuhumyin hundoi, po opeschanen-nomu beregu, 9 August 1947, *A.A. Yunatov* (LE); Gobi-Altaiskii aimak, Altai somon, Dzhungarskaya Gobi, razrushennyi khrebet, mezhdu Adzhi-Bogdo i Altaem, po lozhbinam, 10 August 1947, *A.A. Yunatov* (LE); Ubsunurskii aimak, Under-Hangai somon, sev. makrosklon Hanhueya, v 32 km k YuV ot Barun-Turun somona, polynno-raznotravno-tipchakovaya step, elev. 1390 m, 24 July 1973, *D. Banzragg*, *Z.V. Karamysheva*, *Munhbayar*, *C. Cegmid* (LE).

China. Sinczyan-Uigurskaya avtonomnaya oblast, Sev. Zap. Dzhungariya. Vost. shleify khr. Saur, v 60 km sev. Hosh-Tologoi (na r. Hobuk) po doroge na Altai, gornaya tipchakovaya step, 4 July 1959, *A.A. Yunatov*, *Yuan I-fen* (LE).

## ﻿Conclusion

During our study we confirmed the occurrence of 17 species of genus *Festuca* in the AM. *Festucabrevissima* is a new record to the flora of the AM and at present, its localities in the region are the southernmost within its distribution range. Our revision showed that *F.lenensis* and *F.richardsonii* are not component of the AM flora. All the available specimens of *F.lenensis* from the AM were identified as *F.tschujensis*, whereas specimens of *F.richardsonii* previously recorded for the AM were identified as *F.rubra*. We found also that *F.oreophylla* was previously listed in the flora of the AM as a synonym of *F.musbelica*, however, *F.musbelica* is the earlier legitimate name. Finally, we did not confirm Chusovljanov’s assumption (Chusovljanov 2003) on the presence of *F.kemerovensis* in the AM.

In accordance with our morphological and molecular (Kriuchkova et al. 2023) studies we have revealed three complexes of closely related taxa: *F.kurtschumica*–*F.kryloviana*; *F.sphagnicola*–*F.kuprijanovii*–*F.ovina*; and *F.musbelica*–*F.valesiaca.* Nevertheless, to resolve whether they are separate or conspecific species, further studies including more advanced molecular, morphological, and cytological analyses are needed on the above mentioned complexes of taxa from the entire area of their distribution.

## ﻿Additional information

### Author ORCIDs

Polina D. Gudkova https://orcid.org/0000-0002-6537-8018

Elizaveta A. Kriuchkova https://orcid.org/0000-0003-3704-1730

Alexander I. Shmakov https://orcid.org/0000-0002-1052-4575

Marcin Nobis https://orcid.org/0000-0002-1594-2418

### Data availability

All of the data that support the findings of this study are available in the main text.
